# Purinergic Signalling: Therapeutic Developments

**DOI:** 10.3389/fphar.2017.00661

**Published:** 2017-09-25

**Authors:** Geoffrey Burnstock

**Affiliations:** ^1^Autonomic Neuroscience Centre, University College Medical School London, United Kingdom; ^2^Department of Pharmacology and Therapeutics, The University of Melbourne, Melbourne VIC, Australia

**Keywords:** ATP, adenosine, CNS diseases, peripheral diseases, infection, inflammation

## Abstract

Purinergic signalling, i.e., the role of nucleotides as extracellular signalling molecules, was proposed in 1972. However, this concept was not well accepted until the early 1990’s when receptor subtypes for purines and pyrimidines were cloned and characterised, which includes four subtypes of the P1 (adenosine) receptor, seven subtypes of P2X ion channel receptors and 8 subtypes of the P2Y G protein-coupled receptor. Early studies were largely concerned with the physiology, pharmacology and biochemistry of purinergic signalling. More recently, the focus has been on the pathophysiology and therapeutic potential. There was early recognition of the use of P1 receptor agonists for the treatment of supraventricular tachycardia and A_2A_ receptor antagonists are promising for the treatment of Parkinson’s disease. Clopidogrel, a P2Y_12_ antagonist, is widely used for the treatment of thrombosis and stroke, blocking P2Y_12_ receptor-mediated platelet aggregation. Diquafosol, a long acting P2Y_2_ receptor agonist, is being used for the treatment of dry eye. P2X3 receptor antagonists have been developed that are orally bioavailable and stable *in vivo* and are currently in clinical trials for the treatment of chronic cough, bladder incontinence, visceral pain and hypertension. Antagonists to P2X7 receptors are being investigated for the treatment of inflammatory disorders, including neurodegenerative diseases. Other investigations are in progress for the use of purinergic agents for the treatment of osteoporosis, myocardial infarction, irritable bowel syndrome, epilepsy, atherosclerosis, depression, autism, diabetes, and cancer.

## Introduction

Purinergic signalling, i.e., nucleotides as extracellular signalling molecules, was proposed in 1972 (Burnstock, 1972). However, this concept was not well accepted until the 1990’s when receptor subtypes for purines and pyrimidines were cloned and characterised, which includes four subtypes of the P1 (adenosine) receptor, seven subtypes of P2X ion channel receptors, and eight subtypes of the P2Y G protein-coupled receptor (Ralevic and Burnstock, 1998). Early studies were largely concerned with the physiology, pharmacology and biochemistry of purinergic signalling (Burnstock, 2007). Adenosine 5′-triphosphate (ATP) is a cotransmitter with classical transmitters in both the peripheral and central nervous systems. In addition, purines are powerful extracellular messengers to non-neuronal cells, including secretory, exocrine and endocrine, endothelial, immune, musculo-skeletal and inflammatory cells (Burnstock and Knight, 2004). Purinergic signalling is rapid in neurotransmission, neuromodulation and in secretion, but is also long-term in proliferation, differentiation, migration and death in development and regeneration (Burnstock, 2016f).

More recently, the focus has been on the pathophysiology and therapeutic potential of both P1 (Chen et al., 2013; de Lera Ruiz et al., 2014; Layland et al., 2014; Liu and Xia, 2015; Borea et al., 2016) and P2 (Burnstock and Kennedy, 2011; Bartlett et al., 2014; Ford et al., 2015; Burnstock, 2016e) receptors. Reviews focussed on different aspects of purinergic pathophysiology are also available, including inflammatory and immune disorders (Arulkumaran et al., 2011; Junger, 2011; Hansson et al., 2016); cancer (Burnstock and Di Virgilio, 2013; Di Virgilio and Adinolfi, 2017); gout and fibrosis (Gicquel et al., 2017); P2X7 receptors (R) as therapeutic targets (Romagnoli et al., 2008); medicinal chemistry of purinoceptors (Jacobson and Muller, 2016); pain (Burnstock and Sawynok, 2010; Alves et al., 2013; Kuan and Shyu, 2016; Sawynok, 2016) and adenosine kinase inhibitors (Kowaluk and Jarvis, 2000). A number of purine-related compounds have been patented. Therapeutic developments for disorders of different systems in the body will now follow. Reviews concerned with the early literature will be quoted, so the focus of this review will be concerned largely with the most recent findings.

## Disorders of the Central Nervous System (CNS)

Investigations into purinergic signalling and its roles in disorders of the CNS have been reported, for instance following surgery, stroke, accidents and ischemia, neurodegenerative diseases (such as Parkinson’s, Alzheimer’s and Huntington’s diseases), multiple sclerosis (MS), amyotrophic lateral sclerosis (ALS), epilepsy and neuropsychiatric disorders (including schizophrenia, depression and anxiety). Reviews covering this topic are available (Burnstock, 2008b; Burnstock et al., 2011), including the recent attention to the development of centrally penetrant P2X7R antagonists for the treatment of CNS disorders (Burnstock and Verkhratsky, 2012; Puchalowicz et al., 2014; Sperlagh and Illes, 2014; Burnstock, 2015b; Cisneros-Mejorado et al., 2015b).

### Neurodegenerative Diseases

Neurodegeneration in the CNS is associated with inflammation and damage to both neurons and glia (Rama Rao and Kielian, 2015). Reviews are available that include coverage of early papers concerned with neurodegenerative diseases (Puchalowicz et al., 2014; Santiago et al., 2014; Fasullo and Endres, 2015; Förster and Reiser, 2015; Burnstock, 2016a; Fumagalli et al., 2016). Recent attention has been directed toward the use of P2X7R antagonists for the treatment of neurodegenerative diseases (Metzger et al., 2017). However, adenosine acting via A_2A_R has also been claimed as a promising therapeutic agent for the prevention and treatment of neurodegenerative diseases (Cunha, 2016; Harmse et al., 2016; Olatunji et al., 2016).

#### Alzheimer’s Disease (AD)

There is progressive cognitive impairment in AD, with prominent deficits in short term memory. The potential of purinergic drugs for the treatment of AD has attracted much interest in recent years. Previous studies have led to the proposal that both P2X7R and P2Y_4_R antagonists are potential therapeutic targets for the treatment of AD (Erb et al., 2015; Miras-Portugal et al., 2015; Woods et al., 2016). It has been suggested that the blockade of P2Y_1_R may have therapeutic potential against cognitive disturbances in AD (Guzman and Gerevich, 2016). β-Amyloid increased release of ATP, which potentiated excitatory synaptic activity via P2XR, effects that were blocked by P2X antagonists (Sáez-Orellana et al., 2016). The glycosylphosphatidylinositol-anchored prion protein binds to and modulates the expression of P2X4R, which may be involved in AD (Carneiro et al., 2016). Recently, a potential role has been proposed for ATP-sensitive potassium channel (K_ATP_) modulation as a therapeutic strategy against AD (Salgado-Puga et al., 2017).

Adenosine A_3_R agonists suppress amyloid-β protein precursor internalisation and amyloid-β generation (Li S. et al., 2015). Hippocampal adenosine A_2A_R up-regulation is necessary to trigger memory dysfunction in AD (Cunha, 2015).

#### Parkinson’s Disease (PD)

The involvement of adenosine A_2A_R in PD and their interactions with dopamine receptors has attracted most attention (see Jenner, 2014). A_2A_R antagonists have been proposed for the treatment of PD (Jenner, 2014; Mori, 2014; Pinna, 2014; Navarro et al., 2016). K_ATP_ (Dragicevic et al., 2015) and P2X1R (Gan et al., 2015; Navarro et al., 2016; Yang Z. et al., 2016) in PD have also been reported, perhaps indicating new therapeutic targets. Clinical trials istradefylline, an A_2A_R antagonist, have taken place and it may have a beneficial effect in conjunction with commonly used anti-Parkinson’s therapies (Tao and Liang, 2015; Uchida et al., 2015; Vorovenci and Antonini, 2015), although in a later study, istradefylline was shown to enhance amyloid-β generation and γ-secretase activity (Lu et al., 2016). Oligomerisation kinetics of A_2A_ and dopamine D_2_R have important implications for PD (Casadó-Anguera et al., 2016; Ferré et al., 2016; Guixà-González et al., 2016). Clinical trials for A_2A_ antagonists for PD are assessed in a review (Navarro et al., 2016). A_2A_R inhibition stopped rotenone-induced motor impairment in a rat model of PD (Fathalla et al., 2016). A_1_ as well as A_2A_R antagonists were recommended as promising candidates for treatment of PD in a recent paper (Essawy et al., 2017).

P2X7R antagonists have also been implicated in this disease (Jörg et al., 2014; Wang et al., 2017). A P2X7R antagonist, brilliant blue G, was recently shown to be protective in a lipopolysaccharide (LPS) animal model of PD (Wang et al., 2017).

#### Huntington’s Disease (HD)

Earlier studies reported that A_2A_R agonists could be therapeutically useful for HD and that P2X7R antagonists inhibited neuronal apoptosis and attenuated body weight loss and motor co-ordination deficit in HD patients. Adenosine A_2A_R inhibition reversed working memory deficits of HD at early stages models and it was proposed that A_2A_R antagonists may therapeutically reverse the cognitive deficits in HD patients (Li W. et al., 2015). In earlier publications, A_1_R agonists were suggested to be therapeutic targets (Ferrante et al., 2014; Lee and Chern, 2014). It has been claimed that K_ATP_ channels may be potential targets for treatment of HD (Gupta and Sharma, 2014). Inhibition of equilibrative nucleoside transporter 1 enhances the adenosine level and may be a potential therapeutic approach for treating HD (Kao et al., 2017).

#### Amyotrophic Lateral Sclerosis

Involvement of A_2A_R, P2X4R and P2X7R in ALS has been reported (Volonté et al., 2016). A_1_R have also been implicated in ALS (Nascimento et al., 2015). Preconditioning with latrepirdine, an adenosine 5′-monophosphate (AMP)-activated protein kinase activator, was beneficial in the SOD1 mouse model of ALS (Coughlan et al., 2015). A_2A_R activation facilitated neuromuscular transmission in SOD1 mice during the pre-symptomatic but not symptomatic phase of the disease (Nascimento et al., 2014). Pharmacological inhibition of A_2A_R protects against degeneration of spinal motor neurons in the mouse SOD1G93A ALS model (Ng et al., 2015).

P2X7R expression was significantly reduced, leading to Ca^2+^ disturbances in peripheral blood mononuclear cells in ALS patients (Liu J. et al., 2016). Low concentrations of endogenous ATP acting on P2X7R induced motor neuron death (Gandelman et al., 2013). Spinal cord pathology was reduced by P2X7R inhibitors in the mouse SOD1 ALS model (Apolloni et al., 2014).

#### Multiple Sclerosis

P2X7R and the P2YR-like GPR17 are involved in MS (Plemel et al., 2014; Burnstock, 2015c; Ou et al., 2016). There is upregulation of ecto-5′-nucleotidase (CD73) in experimental autoimmune encephalomyelitis, which is an MS animal model (Lavrnja et al., 2015). Genetic variants in P2X7R affect susceptibility to MS (Gu et al., 2015; Sadovnick et al., 2017).

### Brain Injury, Neuroprotection, and Neuroregeneration

#### Brain Injury

The importance of inflammation in the responses to brain injury has been reviewed and the involvement of P1R and P2R (Fiebich et al., 2014; Beamer et al., 2016) and of P2X7R in particular (Burnstock, 2016b; Giuliani A.L. et al., 2017). The roles of purinergic signalling in neurodegeneration as a consequence of brain injury, neuroprotection and neuroregeneration have been discussed in reviews (Hu et al., 2014; Burnstock, 2016a). Ectonucleotidase activities and nucleotide levels in serum are altered by brain stab injury (Parabucki et al., 2014; Laketa et al., 2015). P2X7R antagonists appear to be effective as a treatment for radiation injury (Xu et al., 2015). Astrocytic *p*-connexin 43 stimulates neuronic autophagy through P2X7R activation in the hippocampus, resulting in brain injury-induced cognitive deficit repair (Sun L. et al., 2015). P2X7R antagonists have been implicated as a novel target to prevent secondary neurological injury after traumatic brain injury (Kimbler et al., 2012) and after spinal cord injury (Peng et al., 2009). P2X7R antagonists could be therapeutically effective to treat stroke patients (Kuan et al., 2015). P2X7R stimulation in acute ischaemic stroke is beneficial by restricting early oedema formation and perhaps by modulating responses of glia (Kaiser et al., 2016). Hypoxic-ischaemic brain injury increases intracellular Ca^2+^ in oligodendrocytes, which is partly mediated by P2X7R (Fern et al., 2014). P2X7R antagonists may provide a new target for the treatment of cerebral ischaemia (Bai and Li, 2013; Yu Q. et al., 2013) and for the prevention of ischaemic damage to oligodendrocytes (Domercq et al., 2010). P2X7 antagonists or inhibition of pannexin-1 channels reduced brain damage following ischaemia (Cisneros-Mejorado et al., 2015a; Mahi et al., 2015).

Activation of upregulated P2Y_1_R results in neuroblast migration to sites of brain damage (Cao et al., 2015). Microglial P2Y_12_R activation follows neuronal injury (Swiatkowski et al., 2016). A_2A_R activation was suggested for the treatment of brain injury and subsequent neuroinflammation (Dai and Zhou, 2011). A_2A_R on cells derived from bone marrow modulate white matter lesions following chronic cerebral hypoperfusion (Ran et al., 2015). Diadenosine tetraphosphate (Ap_4_A) may be a good candidate for traumatic spinal cord injury treatment (Reigada et al., 2017). Adenosine kinase facilitated astrogliosis after traumatic brain injury and its inhibition in reactive astrocytes ameliorated astrogliosis-induced cell death (Jin et al., 2016).

ATP and adenosine concentrations in the brain were significantly raised during brain ischaemia to stimulate P1R and P2R (Cisneros-Mejorado et al., 2015b; Pedata et al., 2016). P2X4R expressed by microglia are involved in post-ischaemic brain inflammation (Cheng et al., 2014). P2X4R are required for neuroprotection via ischemic preconditioning (Ozaki et al., 2016). Neuronal K_ATP_ channels play a role in hypoxic preconditioning where they decrease neonatal hypoxic-ischaemic brain injury; it has been proposed that openers of K_ATP_ channels may prove to be therapeutically beneficial (Sun H.S. et al., 2015). Intranasal administration of guanosine reduced ischaemic brain damage in rats (Ramos et al., 2016). Ambiguously, both A_2A_R agonists and antagonists may protect against ischaemic brain injury (Pedata et al., 2014). A_1_R contribute to immune responses following neonatal hypoxic ischaemic brain injury (Winerdal et al., 2016). A valuable review of the involvement of P1R, P2XR and P2YR in brain ischemia is available (Pedata et al., 2016).

#### Neuroprotection and Neuroregeneration

This topic is explored in recent reviews (Rodrigues et al., 2015; Burnstock, 2016a; Illes et al., 2016). Inhibition of P2XR and P2YR as well as activation of P1R following ATP breakdown to adenosine released from CNS cells, have been shown to be neuroprotective. Activation of the pannexin 1/P2X7R complex contribute to the neuroprotection that occurs after ischaemic pre- and post-conditioning (Mahi et al., 2015). Docosahexaenoic acid in the diet is thought to be a purinergic modulator via P2XR where it protects against neurodegenerative diseases (Molz et al., 2015). Neuroprotection mediated by microglia is associated with P2X7R activation and release of tumour necrosis factor-α (Masuch et al., 2016). Blockade of P2X7R provides neuroprotection against stroke, traumatic brain injury and subarachnoid haemorrhage (Zhao H. et al., 2016). P2X7R antagonists improve recovery after spinal cord injury (Wang et al., 2004). From a recent study on P2Y_2_R knockout (KO) mice and murine cell lines, it was concluded that P2Y_2_R play a neuroprotective role in neurological disorders, especially AD (de Diego-Garcia et al., 2017). Neuroprotection is claimed to be mediated by P2Y_13_ nucleotide receptors in neurons (Pérez-Sen et al., 2015). Activation of P2Y_2_R evokes regeneration of glial cells and nerves and the P2YR-like GPR17 evokes oligodendrocyte regeneration. Neural stem cell activation leads to neuroregeneration, probably via P2X4R and P2X7R. Reviews focused on the roles played by P2X4R and P2X7R (Miras-Portugal et al., 2015) and A_2A_R (Ribeiro et al., 2016) in neurodegeneration and neuroprotection have been published.

### Psychiatric Disorders

Several antipsychotic drugs (e.g., chlorpromazine, fluspirilene, and haloperidol) were found to antagonise responses mediated by P2XR. These antipsychotic drugs act therapeutically by inhibiting dopaminergic hyperactivity by suppressing P2X-mediated effects (see Burnstock, 2015b). Brain penetrant P2X7R antagonists are being developed as drug targets for psychiatric diseases (Bhattacharya and Biber, 2016). Reviews about purinergic signalling in psychiatric disorders, such as addiction, depression, schizophrenia, bipolar disorder and autism, have been previously published (Yamada et al., 2014; Lindberg et al., 2015; Krügel, 2016). The possibility has been raised that P1R agonists might be beneficial in the therapy of psychiatric disorders (Cieslak et al., 2016).

#### Schizophrenia

Adenosine neuromodulation in schizophrenia has received the most attention (see Rial et al., 2014; Ciruela et al., 2015; Turcin et al., 2016). Deletion of A_2A_R from astrocytes interferes with glutamate homeostasis resulting in cognitive and psychomotor impairment in schizophrenia (Matos et al., 2015). It has been suggested that P2X7R and pannexin 1 channels are involved (see Burnstock and Verkhratsky, 2012; Avendano et al., 2015).

#### Bipolar Disorder

P2X7R, mediating neuroinflammation via microglial activity, contribute to bipolar disorder (see Gubert et al., 2013, 2016; Barron et al., 2014). Serum concentrations of uric acid increase in different clinical phases of bipolar disorder (Albert et al., 2015) and in those patients treated with lithium (Muti et al., 2015). The purinergic system may become dysregulated during manic episodes and it has been proposed that raised uric acid levels could be a useful indicator of bipolar disorder manic phases (Bartoli et al., 2017a). Purinergic modulators that reduce levels of uric acid may be therapeutically beneficial (Bartoli et al., 2017b). However, a decrease in serum adenosine levels in bipolar disorder patients was reported (Gubert et al., 2016).

#### Depression and Anxiety

Reviews describing the roles of both P1 (A_1_ and A_2A_) and P2X7R in mood disorders are available (Yamada et al., 2014; Ortiz et al., 2015). Antidepressant effects of P2X7 antagonists have been reported (Pereira et al., 2013; Zhang K. et al., 2016). P2X2R in the medial prefrontal cortex mediate the antidepressant-like actions of ATP released from astrocytes (Cao et al., 2013). Brilliant blue G, a P2X7R antagonist, had antidepressant and anti-inflammatory actions in mice after LPS administration (Ma et al., 2014). The P2X7R antagonist, A-804598, affected neuroimmune and behavioural features of stress (Catanzaro et al., 2014). Stress-related mood disorders activate the inflammasome via release of ATP and stimulation of P2X7R (Iwata et al., 2016). Co-expression of wild-type P2X7R with the polymorphism variant Gln460Arg alters receptor function associated with mood disorders (Aprile-Garcia et al., 2016). Increased K_ATP_ channel activity due to a mutation led to reduced anxiety in mice (Lahmann et al., 2014). K_ATP_ channels are involved in the pathogenesis of depression and may be a therapeutic target for this disorder (Fan et al., 2016). Altered levels of several ATP-dependent chromatin remodelling factors may be linked to high trait anxiety (Wille et al., 2016).

A_2A_R antagonists were reported to have antidepressant activity (Yamada et al., 2014). Creatine and ketamine had antidepressant effects, probably mediated by activation of A_1_R and A_2A_R (Cunha et al., 2015). Caffeine, acting as an A_2A_R antagonist, prevented depression triggered by chronic stress (Kaster et al., 2015; Dziubina et al., 2016). Striatal and extrastriatal A_2A_R in the forebrain regulate fear responses in mice (Wei et al., 2014). A_2A_R mediated increased interleukin (IL)-1β in the brain contributed to anxiety (Chiu et al., 2014). Fear and anxiety in a mouse model of post-traumatic stress disorder were alleviated by administration of a derivative of adenosine, WS0701 (Huang et al., 2014). A_1_R agonists have been used to treat anxiety, but have troublesome side-effects so positive allosteric modulators have been developed as potent anxiolytic agents (Vincenzi et al., 2016). An antidepressant-like effect of inosine in mice has been reported (Gonçalves et al., 2017).

#### Autism

Adenosine in addition to suramin, a non-selective ATP antagonist, are reported to improve behaviour in autistic individuals (Masino et al., 2013; Naviaux et al., 2014, 2015; Hamidpour et al., 2016).

#### Addiction

Targeting A_2A_R may offer strategies for combating drug addiction. Striatopallidal A_2A_R signalling in the dorsomedial striatum has been suggested as a therapeutic target in drug addiction by reducing habit formation (Li et al., 2016). Caffeine potentiates the addictive effects of drugs of abuse, including cocaine and amphetamine derivatives (Ferrée, 2016).

Treatment of rodents with a P2X3R antagonist diminished opioid tolerance (Tai et al., 2010) and tolerance to morphine-induced antinociception (Ma et al., 2015). Lead-induced neuroinflammation via P2X7R may be responsible for the intensification of morphine tolerance (Baranowska-Bosiacka et al., 2016). Opiate-induced changes in brain adenosine levels may be associated with opiate addiction and withdrawal (Wu et al., 2013).

Adenosine, acting via A_2A_R, regulates addiction induced by cocaine. Women are more sensitive to cocaine and are therefore more vulnerable to becoming addicted. Adenosine antagonists may be an effective treatment (Broderick and Malave, 2014). A_2A_-D_2_ receptor-receptor interactions in the dorsal striatum are differentially affected by cocaine, which contributes to compulsive drug seeking (Pintsuk et al., 2016).

Interactions between striatal A_2A_ and glutamate (mGlu5) receptors modulate the drug-seeking effects of methamphetamine (Wright S.R. et al., 2016). Methamphetamine produces alterations in adenosine receptor expression in the nucleus accumbens (Kavanagh et al., 2015). A_2A_R antagonism in dorsomedial striatum reduces methamphetamine addiction (Furlong et al., 2015). Adenosine A_2A_R integrate the rewarding and motivational behaviours of methamphetamine (Chesworth et al., 2016). Behavioural sensitisation provides a model in animals for drug craving that underlies human addiction and sensitisation to amphetamine was reduced by P2Y_1_R antagonists in the mesocortico-limbic dopaminergic system (Krügel et al., 2013).

A_2A_R agonist treatment can help counteract nicotine addiction (Jastrzebska et al., 2014). A_2A_R are a potential target for the treatment of alcohol abuse (Micioni Di Bonaventura et al., 2012; Houchi et al., 2013). Regulation in adenosine signalling in striatal circuits in alcohol addiction was reviewed (Nam et al., 2013). A_1_R signalling contributes to the regulation of basolateral amygdala excitability and to the pathophysiology of alcohol addiction (Rau et al., 2014). A review of alcohol addiction discusses the role of adenosine (Michalak and Biala, 2016). P2X4R modulate synaptic signalling associated with alcohol addiction (Franklin et al., 2014; Khoja et al., 2016).

### Epilepsy

Early focus was on the role of P1R in epileptic seizures, but P2X4R and P2YR, but especially P2X7R antagonists have been recently explored as neuroleptic agents (see Burnstock, 2015b; Engel et al., 2016; Rassendren and Audinat, 2016; Beamer et al., 2017; Cieslak et al., 2017). Seizure-induced increases in microglial process numbers were reduced and kainate-induced seizure behaviours were exacerbated in P2Y_12_ KO mice (Eyo et al., 2014). Antiepileptic consequences of deep brain stimulation may be mediated by P1R activation (Miranda et al., 2014). P2X3R expression was upregulated in epileptic rats and humans and it was postulated that P2X3R antagonists might be therapeutically effective (Zhou X. et al., 2016). The release of adenosine and ATP during on-going epileptiform activity was measured with microelectrode biosensors (Frenguelli and Wall, 2016). ATP levels were increased during epileptic seizures, which were not of neuronal origin (Lietsche et al., 2016). There is an insightful Editorial about purinergic signalling-induced neuroinflammation in epilepsy (Engel, 2016).

A role for post-transcriptional control of the P2X7R expression has been proposed and therapeutic targeting of microRNA-22 was suggested to prevent development of epilepsy and inflammation (Jimenez-Mateos et al., 2015). Purinergic signalling, via P2X7R, regulates neonatal seizures associated with hypophosphatasia, an inherited metabolic bone disease characterised by spontaneous seizures (Sebastián-Serrano et al., 2016). P2X7R antagonists were effective against hypoxia-induced neonatal seizures in mice (Rodriguez-Alvarez et al., 2017).

### Migraine

The role of ATP in migraine was initially suggested to involve vascular events (see Burnstock and Verkhratsky, 2012; Haanes and Edvinsson, 2014). Later, P2X3R in nociceptive brain areas, including the thalamus and trigeminal nucleus, were investigated and their interaction with P2Y_1_R in trigeminal neurons (Hullugundi et al., 2014; Marchenkova et al., 2016). The therapeutic potential of antagonists to P2X7R for migraine treatment has been proposed (Gölöncsér and Sperlágh, 2014), as well as P2X3R and P2X2/3R antagonists (Kilinc et al., 2015; Yegutkin et al., 2016). Reviews have been published about the roles and therapeutic potential of purinergic signalling in the aetiology of migraine (Cieslak et al., 2015; Yegutkin et al., 2016).

### Neuropathic Pain

P1R and P2R involvement in neuropathic pain has been discussed in reviews (Burnstock and Sawynok, 2010; Burnstock et al., 2011; Burnstock, 2014a, 2016c). The discovery by Inoue and colleagues that antagonists to P2X4R on microglia are effective against neuropathic pain was particularly important (see Tsuda, 2016). Antagonists to P2X7R and P2Y_12_R also act on microglia to reduce neuropathic pain (see Tsuda and Inoue, 2016; Tsuda, 2017). A_3_R agonists delay the development of neuropathic pain (Janes et al., 2016). Glial P2Y_2_R are potential targets for the management of trigeminal-related pain (Magni et al., 2015). Pannexin 1 and P2X7R interactions have been suggested to play a role in chronic pain (Bravo et al., 2015). P2X7R antagonists have been recommended for the treatment of central post-stroke pain (Kuan et al., 2015). A review discusses the use of P2XR subtype antagonists for the treatment of central neuropathic pain (Kuan and Shyu, 2016). Purinergic signalling in the spinal dorsal horn and anterior cingulate cortex appear to be involved in neuropathic pain (Tsuda et al., 2017). P2X3 and P2X2/3R blockade has also been claimed to reduce chronic pain (Cantin et al., 2012; Xu et al., 2012; Giniatullin and Nistri, 2013).

### Brain Tumours

Neuroblastoma, a rare childhood tumour, expresses P2X7R, which mediate proliferation. The P2X7R appears to be a regulator of neuroblastoma metabolic activity, angiogenesis and growth and may be a target for neuroblastoma treatment (Amoroso et al., 2015; Gomez-Villafuertes et al., 2015). The action of the antitumor agent, temozolomide, was increased by the antiproliferative actions of P2X7R agonists and antagonists to A_3_R and P2Y_1_R on human glioblastoma (D’Alimonte et al., 2015). P2X4R may also be involved in human neuroblastoma (Gualix et al., 2015).

P2X7R were over-expressed in human malignant gliomas and P2X7R antagonists decreased tumour cell numbers (Fang et al., 2015; Morrone et al., 2016; McLarnon, 2017). Extracellular nucleotides control glioma growth via P2X7R and P2Y_6_R activation (Braganhol et al., 2015). P2X7 and A_2A_R activation leads to release of cytokines by macrophages, which was prevented by antagonists to these receptors (Bergamin et al., 2015). P2X7R antagonists blocked the cell cytotoxicity caused by irradiation for glioma (Gehring et al., 2015). Purine nucleoside phosphorylase is released from rat C6 glioma cells, contributing to the purinergic system homeostasis and exhibiting a pathophysiological role (Giuliani P. et al., 2017).

It was claimed that K_ATP_ channels are associated with tumorigenesis of human glioma (Ru et al., 2014). A_3_R blockade enhances the actions of antitumour drugs used against human glioblastoma stem-like cells (Torres A. et al., 2016). A_1_ and A_2B_R also sensitise glioblastoma stem cells to chemotherapy (Daniele et al., 2014). P2Y_2_R interactions with caveolin-1 represents a novel target for human astrocytoma cells (Martinez et al., 2016).

### Sleep Disorders

P2Y_11_R appear to be associated with narcolepsy (Kornum et al., 2011). Reduced endothelial dilation to ATP in cerebral arteries occurred in a rat model of obstructive sleep apnoea (Crossland et al., 2013). Adenosine is a key player in the regulation and maintenance of sleep-wake dependent neural activity changes, where dysregulation can lead to sleep-wake disorders (Holst et al., 2016). The A_2A_R antagonist, SCH58261, overcame the blood–brain barrier dysfunction as a result of sleep restriction (Hurtado-Alvarado et al., 2016).

## Cardiovascular Diseases

Reviews about this topic are available (Erlinge and Burnstock, 2008; Headrick et al., 2013; Burnstock and Ralevic, 2014; Burnstock, 2015a; Burnstock and Pelleg, 2015; Ralevic, 2015; Sousa and Diniz, 2017).

### Heart Diseases

#### Heart Failure

In chronic heart failure adenosine accumulates, probably as a result of lowered adenosine deaminase (ADA) gene expression and raised CD73 activity. Adenosine therapy mediated by A_1_R and A_3_R is cardioprotective for chronic heart failure (Greene et al., 2016; Voors et al., 2017). A_1_R agonists attenuate cardiac hypertrophy and prevent heart failure in a mouse model (left-ventricular pressure-overload) and in a rat model (neonatal cardiac myocyte) (Chuo et al., 2016). A_2B_R agonists exert stronger cardioprotective effects against cardiac ischaemia/reperfusion injury compared to A_2A_R activation in rats (Ke et al., 2015). CD73 and A_2B_R agonists have been considered as therapeutic agents for myocardial ischaemia. Genetic deletion of CD39 results in increased myocardial ischaemia-reperfusion injury (Smith et al., 2016). Inflammatory responses initiated during ischemia-mediated immune injury may be regulated by adenosine (Boros et al., 2016).

Early studies were concerned with the role of adenosine in ischaemic and reperfusion injuries. However, there is also interest in the role of ATP. Application of ATP, prior to or just after cardiac ischaemia is cardioprotective (Ren et al., 2016). ATP released from the ischaemic myocardium causes reflex responses mediated by cardiac sympathetic afferent nerves (Dong et al., 2016). P2YR are important therapeutic targets in myocardial protection during ischemia/reperfusion (Djerada et al., 2017). P2Y_6_R could be a therapeutic target to regulate cardiac hypertrophy (Clouet et al., 2016). There is increased expression of P2Y_2_ and P2X1R in the hearts of rats with congestive heart failure.

P2X4R are needed for neuroprotection via ischemic preconditioning (Ozaki et al., 2016). P2X3R expression increased in dorsal root ganglion (DRG) and superior cervical ganglia neurons, resulting in exaggerated sympathoexcitatory reflexes. NONRATT021972 siRNA decreases the upregulation of P2X7R and improves cardiac function after myocardial ischemia (Tu et al., 2016). Mitochondrial K_ATP_ channels provide protection against myocardial ischemia/reperfusion injury (Wang et al., 2015; Shimizu and Calvert, 2016). K_ATP_ channels maintain high energy phosphates and myocardial perfusion in heart failure (Jameel et al., 2016).

#### Myocardial Infarction

A clinical trial (Acute Myocardial Infarction Study of Adenosine) concluded that infusion for 3 h of adenosine resulted in a reduction of infarct size (Yetgin et al., 2015; Bulluck et al., 2016). Protection against myocardial infarction was mediated by A_1_R in the rabbit heart. The A_3_R agonist IB-MECA produced cardioprotection against myocardial infarction (Tian et al., 2015). Microglial P2X7R in the rat hypothalamic paraventricular nuclei regulate the sympathoexcitatory responses in acute myocardial infarction (Du D. et al., 2015). Loss of mouse P2Y_4_R protects against myocardial infarction (Horckmans et al., 2015). CD39 reduced infarct size following ischaemia-reperfusion injury (Smith et al., 2016). P2Y_12_R antagonists have been recommended for long-term protection of patients, post-myocardial infarction (Alexopoulos et al., 2016). The K_ATP_ channel opener, natakalim, improves ventricular remodelling of congestive heart failure after myocardial infarction (Jin, 2016).

#### Atrial Fibrillation

Adenosine reduces post-operative atrial fibrillation (AF). Up-regulation of A_2A_R involves abnormal calcium handling in AF. Prevention of A_2A_R activation in patients with AF may sustain uniform beat-to-beat responses at higher beating frequencies (Molina et al., 2016). Adenosine-guided pulmonary vein isolation following a randomised clinical trial was recommended to treat paroxysmal AF (Macle et al., 2015), although this has been queried in a more recent clinical trial (Ghanbari et al., 2016). ATP-induced AF has also been investigated (Hasebe et al., 2016). The efficacy of the P2Y_12_ antagonists ticagrelor and prasugrel are not affected in AF (Ondrakova et al., 2016). The roles of adenosine and ATP in atrial arrhythmias and fibrillation have been discussed (Jared Bunch, 2015; Belhassen and Michowitz, 2016).

#### Supraventricular Tachycardia

Acute therapy by ATP for paroxysmal supraventricular tachycardia was used in the late 1940’s. In patients with paroxysmal supraventricular tachycardia bolus injection of Adenocard (adenosine) is clinically prescribed to slow conduction time via the atrioventricular node, via A_1_R (Sachdeva and Gupta, 2013). Treatment of paroxysmal supraventricular tachycardia by adenosine and ATP is discussed (Lerman, 2015). To provoke vasovagal reaction in syncope patients ATP and adenosine are administered together with the head-up tilt table test (Fragakis et al., 2015).

#### Cardiomyopathy

Cardiomyopathy can be an inherited disease, but can occur as a result of vitamin B deficiency, amyloidosis, alcoholism or viral infections. ATP synthase disruption contributes to diabetic cardiomyopathy (Ni et al., 2016). P2X7R involvement in dilated cardiomyopathy has been reported (Martinez et al., 2015).

#### Cardiac Fibrosis and myocarditis

P2Y_11_R agonists reduce cardiac fibrosis (Certal et al., 2015). Extracellular nucleotide regulation of signalling in cardiac fibrosis has been discussed (Novitskaya et al., 2016). The P2X7R antagonist, A740003, reduces experimental autoimmune myocarditis, suggesting a treatment for clinical myocarditis (Zempo et al., 2015).

#### Angina

ATP injections to treat angina pectoris associated with coronary disease were used during the 1940s, while AMP was used to treat angina. ATP treatment for patients with coronary insufficiency was also used early. Intracoronary administration of adenosine results in angina pain. Intracoronary administration of adenosine in patients with unstable angina produced decreased myonecrosis and improved coronary blood flow (Kizilirmak et al., 2015). In vascular pain, which encompasses pelvic and ischaemic pain, migraine and angina, it is thought that release of ATP from endothelial cells during reactive hyperaemia after vasospasm diffuses through the microvascular wall to activate P2X3R on perivascular sensory nerves to send impulses that travel to pain centres in the brain via the spinal cord (Joseph et al., 2015). P2X2/3R expressed on airway nociceptive sensory nerves mediate cardiovascular reflexes in conscious rats (Hooper et al., 2016).

#### Cardiac Transplants

Responses of the transplanted human heart to adenosine show supersensitivity. Protection of cardiac grafts from cold ischaemia/reperfusion injury is caused by donor pretreatment with AMP-activated protein kinase (Yang C. et al., 2016). Treatment with P2XR antagonists prolongs cardiac transplant survival.

### Vascular Diseases

#### Hypertension

It has been proposed in a recent review (Burnstock, 2017) that there are five different ways that purinergic signalling can contribute to the development of hypertension:

(1) ATP released as a cotransmitter from sympathetic nerves together with noradrenaline (NA) contributes, via P2X1R, to the vasoconstriction that results from increased sympathetic vasomotor activity in hypertension. Therefore, P2X1R antagonists should be useful for the treatment of hypertension, especially since there is a substantial increase in ATP relative to NA released from sympathetic nerves in spontaneously hypertensive rats (Brock and Van Helden, 1995; Goonetilleke et al., 2013).
(2) Release of ATP from endothelial cells by shear stress as a result of changes in blood flow acts on P2YR and P2X4R on endothelial cells to release nitric oxide (NO) resulting in vasodilation. Introduction of P2Y_1_, P2Y_2_, and P2X4 agonists would cause increased vasodilation in hypertension.
(3) Brain stem and hypothalamic neurons mediate sympathetic nerve activity. Recent studies show that P2X3R antagonists are antihypertensive, due to reduced sympathetic nerve activity as a result of increased peripheral P2X3R-mediated carotid body chemoreceptor reflexes (Pijacka et al., 2016).
(4) P2X7R in the kidney contribute to the pathophysiology of hypertension and P2X7R antagonists may have promise as clinical antihypertensive agents.
(5) In hypertensive patients adenosine activates the vascular renin-angiotensin system. P1R agonists have been suggested for the treatment of hypertension (Ho M.F. et al., 2016).

Recently, P2Y_6_R were shown to age-dependently promote vascular remodelling in a mouse model, an effect inhibited by MRS2578, suggesting that P2Y_6_R are a therapeutic target for the prevention of age-related hypertension (Sunggip et al., 2017).

#### Atherosclerosis

ATP signalling influences the development of atherosclerosis (Burnstock, 2008a; Ferrari et al., 2015). Endothelial and smooth muscle cell proliferation are promoted by adenosine and ATP in atherosclerosis via P2Y_1_, P2Y_2_, and P1R. In a human model, adenosine, via A_2A_R, modulates foam cell formation (Reiss and Cronstein, 2012). A_2B_ and A_3_ antagonists reduce atherosclerotic plaque development. There are increased concentrations of circulating adenosine 5′-diphosphate (ADP) and ATP in atherosclerosis (Jalkanen et al., 2015). Uridine 5′-triphosphate (UTP), via P2Y_2_R, induces expression of vascular cell adhesion molecule-1 in coronary artery endothelial cells, which leads to the monocyte recruitment associated with atherosclerosis development. Upregulated P2Y_2_R mediate intimal hyperplasia in collared rabbit carotid artery. P2Y_1_R antagonists are a therapeutic target for neointima formation (Liu R. et al., 2015). Endothelial P2X4R play a more important role in intense proliferation in atherosclerosis than P2Y_2_R. ATP, by inducing leukocyte recruitment in mice, contributes to atherogenesis, via P2Y_2_, P2Y_6_, P2X4, and P2X7R. P2X7R are over-expressed in atherosclerosis and P2X7R deficiency leads to less plaque formation (Stachon et al., 2016). CD39 mRNA-coated stents may be a treatment for atherosclerosis (Abraham et al., 2015).

Atherosclerosis of coronary vessels is called coronary artery disease or coronary artery syndrome. P2Y_12_R antagonists combined with aspirin are beneficial for patients with acute coronary syndrome (De Luca et al., 2016; Rollini et al., 2016). A_2A_R on coronary arteries have also been claimed to be involved in coronary artery disease (Gariboldi et al., 2017).

#### Vascular Injury, Angiogenesis and Restenosis

An initiating event in the pathogenesis of vascular diseases is often vascular injury. Injured cells release ATP, which, together with adenosine, evoke endothelial and smooth muscle cell growth, proliferation, migration and death. Adenosine, following breakdown of ATP, is protective against ischaemic injury. P2Y_12_R antagonists are prescribed as prevention against ischaemic stroke (Liu F. et al., 2015). P2Y_2_R mediate regulation of endothelial inflammation and angiogenesis (Gidlöf et al., 2015).

Sustained control of proliferation of endothelial and smooth muscle cells as a result of P1 and P2YR activation during vascular remodelling in restenosis after angioplasty has been reported and therapeutic possibilities are being explored (Burnstock, 2002). Activation of A_2B_R stimulates angiogenesis in human microvascular endothelial cells (Du X. et al., 2015). Adenosine, via A_2A_R, stimulates wound healing and angiogenesis following tissue injury in mice. CD39 administration decreases injury-induced platelet deposition and recruitment of leukocytes and inhibits neointimal hyperplasia. A review discusses the vascular actions of P2XR in renal injury (Howarth et al., 2015).

#### Thrombosis, Inflammation, and Stroke

Nucleotides are extracellular mediators of vascular inflammation and thrombosis. Clopidogrel, a P2Y_12_ antagonist, inhibits aggregation in platelets and is widely prescribed for thrombosis and stroke (Sarafoff et al., 2012). Other P2Y_12_ antagonists have been developed, including ticlopidine, cangrelor, ticagrelor, prasugrel, elinogrel, BX 667, and PSB 0739. P2Y_1_R antagonists also have antithrombotic actions and have been recommended as a complement to current P2Y_12_ anti-platelet strategies. The P2Y_6_R may be a therapeutic target for systemic inflammatory responses. Review articles are available about purinergic signalling in thrombosis and inflammation, including the use of different oral or intravenous P2Y_12_R antagonists (Tang et al., 2015; Nylander and Schulz, 2016; Rollini et al., 2017). P2X7R are pro-thrombotic and genetic KO of the gene for the P2X7R was shown to be protective in a mouse carotid artery thrombosis model (Furlan-Freguia et al., 2011). Adenosine, via A_2A_R and A_3_R, had antithrombotic effects (Cristalli et al., 1994; Hofer et al., 2013).

#### Migraine

Migraine pain involves two distinct cerebrovascular phases: an initial vasoconstriction (with no pain), followed by vasodilation (reactive hyperaemia) associated with pain. A purinergic hypothesis for migraine was proposed in 1981 (Burnstock, 1981). It was suggested that after the initial vasospasm ATP and adenosine (following breakdown of ATP) may mediate the vasodilation during reactive hyperaemia associated with pain. It was also hypothesised that stimulation of P2X3R on sensory nerve terminals located on the adventitia of the cerebral microvasculature by ATP contributed to the migraine pain. Data has also been presented recently that is consistent with the purinergic hypothesis of migraine pain (Yegutkin et al., 2016). P2X3R antagonists have been proposed as potential candidates for migraine treatment (Waeber and Moskowitz, 2003). The non-steroidal anti-inflammatory compound, naproxen, currently in use for migraine pain, blocks P2X3R-mediated responses in trigeminal neurons of the rat. Migraine could involve a chronic disorder of the sympathetic nervous system, where increased release of the sympathetic cotransmitter ATP contributes to the initial vasospasm.

Adenosine may also be involved in migraine. Adenosine infusion resulted in symptoms that were migraine-like and withdrawal of the P1R antagonists theophylline and caffeine also resulted in migraine-like symptoms. Clinical trials with the adenosine uptake inhibitor dipyridamole, that results in increased extracellular adenosine, were halted due to the increase of migraine attacks in all patients. Overactive P2YR on glial cells may contribute to pain transduction during migraine. Reviews have been published about the role of purinergic signalling in the aetiology of migraine and the potential of purinergic compounds (Cieslak et al., 2015; Jacobs and Dussor, 2016).

#### Diabetic Vascular Disease

There is pre-junctional A_1_R-mediated sympathetic neurotransmission and ATP-mediated endothelial vasodilatation in mesenteric arteries of streptozotocin (STZ) diabetic rats (Burnstock and Novak, 2013). There is enhanced A_2A_R-mediated increase in coronary flow in type 1 diabetic mice (Labazi et al., 2016). UTP, ATP and adenosine evoked vasodilation is reduced in the circulation of skeletal muscle of type 2 diabetic patients. Erythrocyte release of ATP is diminished in type 2 diabetics, supporting the view that a defect in the physiology of erythrocytes may contribute to diabetic vascular disease. Erythrocytes are less deformable in type 2 diabetes leading to lowered levels of deformation-induced ATP release. Low erythrocyte ATP release may contribute to the prevention and treatment of diabetic peripheral vascular disease (Richards et al., 2015). P2X7R expression by monocytes might play a role in the pathological changes of type 2 diabetes mellitus (Wu et al., 2015).

#### Aortic Valve Disease

ATP inhibits mineralisation of the aortic valve seen in calcific aortic valve disease (Côté et al., 2012). Increased levels of ectonucleotidase are found in calcific aortic valve disease and inhibition of ectonucleotidase with ARL67156 prevented disease development in rats.

### Blood Cell Diseases

Adenosine is a potential therapeutic target for the prevention and treatment of sickle cell disease (Fu and Davies, 2015). The circulating levels of adenosine are elevated in pregnant women with sickle cell disease (Ashimi et al., 2015). Amyloid-β peptide inhibits ATP release from erythrocytes, suggesting that in AD, vascular amyloid peptide may play a role.

## Diseases of the Airways

Reviews covering the early literature are available (Burnstock et al., 2012a; McGovern and Mazzone, 2014).

Inflammation occurs in most diseases of the airways, including chronic obstructive pulmonary disease (COPD), dyspnea, asthma, cystic fibrosis (CF), allergy, infection and injury. Purine nucleotide release from airway epithelial cells is raised in inflammatory processes and has an important role in the pathophysiology of chronic lung disease. P2X7R are a therapeutic target in lung hypersensitivity reactions seen in chronic inflammatory responses. P2X7R modulate lung inflammatory, fibrotic and functional changes in silicosis, an occupational lung disease (Monção-Ribeiro et al., 2014). P2X4R mediate acute airway inflammation by regulating dendritic cell function (Wiesler et al., 2016). Vagal parasympathetic reflex bronchoconstriction has a role in inflammatory airway disease. Mucin hypersecretion is stimulated in various respiratory diseases and silencing of MUC8 by siRNA increased P2Y_2_R-induced airway inflammation (Cha et al., 2015). P2Y_2_R have also been claimed to downregulate MUC5AC gene expression (Jeong et al., 2016). Nucleotides released during airway inflammation activate P2Y_6_R leading to further release of inflammatory cytokines (Hao et al., 2014). Anti-inflammatory effects of adenosine in the lung have been described involving immune cells.

### Asthma

A role of adenosine in asthma has been considered for many years, because it is a powerful bronchoconstrictor in asthmatic, but not healthy lungs and P1R antagonists have been used for the treatment of asthma (see Cicala and Ialenti, 2013). It was suggested earlier that the bronchoconstriction evoked by adenosine in asthma was as a result of indirect actions by release of leukotrienes, histamine or endothelin (see Burnstock et al., 2012a). In early reports, A_2A_, A_2B_, and A_3_R were all claimed to mediate inhibition of allergic airway inflammation and mast cells were shown to be involved. Expression of adenosine receptors on monocytes from patients with asthma contributes to the progression of the disease (Yuryeva et al., 2015). A_2A_R are involved in the immunological pathogenesis of asthma (Wang et al., 2016).

The roles of nucleotides in asthma has gained attention. In human lung mast cells, ATP is an important modulator of histamine release. Attenuated P2X7R function gives protection from asthma and was suggested to be age related. ATP in sputum was significantly elevated in patients with asthma, which was correlated with the percentage of neutrophils in sputum (Soma et al., 2016). Impaired P2X1R-mediated adhesion in eosinophils from asthmatic patients has been reported (Wright A. et al., 2016). P2Y_6_R activation was effective for treatment of a mouse model of asthma (Chetty et al., 2016).

In allergic asthma, inhalation of allergens, such as pollen spores or house dust mite allergen, or triggers such as viral infection or air pollution, trigger inflammation. For an immune response to be triggered by allergens, activation of dendritic cells is required. In allergic asthmatics, bronchoconstriction following inhaled adenosine was mediated by A_1_R. Selective antagonists to A_2B_R improve inflammatory conditions in allergic asthma (Basu et al., 2017). ATP plays a role in inflammation in allergic asthma by recruitment and activation of inflammatory cells. P2Y_1_R are involved in allergic airway inflammation, probably by regulating maturation of dendritic cells. It has also been proposed that P2Y_2_R in the respiratory epithelium are important sensors for airborne allergens. P2X7R have been implicated in the pathophysiology of allergy-induced lung inflammation. Activating P2X7R on hematopoietic cells, namely eosinophils or dendritic cells, may be a therapeutic approach to treat allergic asthma. Oxatomide, an anti-allergic antihistamine, is claimed to also act as a P2X7R antagonist (Yoshida et al., 2015). P2R act as modulators of rat eosinophil recruitment in allergic inflammation (Alberto et al., 2016). Asthmatic patients exhibit hypersensitivity to aerosolised ATP, but the effects of ATP are not mediated by adenosine (Basoglu et al., 2017).

### Chronic Obstructive Pulmonary Disease (COPD)

COPD emphysema and chronic bronchitis. COPD is caused by gas or noxious particles, particularly in tobacco smoke, trigging a lung inflammatory response that when in the larger airways is called chronic bronchitis while in the alveoli is called emphysema.

The role of adenosine receptors in COPD has been the main emphasis to date. Combined stimulation of A_2B_ and glucocorticoid receptors in epithelial cells of human airways induces genes that have anti-inflammatory potential for COPD (Greer et al., 2013). A_2B_R on human mast cells were claimed to be a strategic target for COPD and inhaled A_2A_R agonists have been used for the treatment of COPD. A_1_, A_2B_, and A_3_R antagonists have also been used to treat COPD (Basu et al., 2017).

ATP is implicated in COPD as well as adenosine. COPD is characterised by up-regulation of ATP in bronchoalveolar lavage fluid, which promotes inflammation and tissue degradation. ATP-induced pulmonary vasodilation occurs in patients with COPD. Activation of P2X7R signalling by cigarette smoke appears to play a role in the pathogenesis of emphysema. Cigarette smoke induces neutrophil ATP release via P2X7 and probably P2Y_2_R activation. Aerosolised ATP exacerbates the symptoms of COPD (Basoglu et al., 2015). A valuable review about purinergic signalling and COPD has been published recently (Pelleg et al., 2016).

### Airway Infections

Antibiotics, including erythromycin, are used widely for the treatment of lower and upper respiratory tract infections. Erythromycin blocks the P2XR-mediated Ca^2+^ influx and could represent one mechanism by which it exerts its effects. The airway epithelium has a role in activating the innate immune response during lung bacterial infections to fight the infection. Over-expression of the ectonucleotidase, CD39, promotes bacteria-induced inflammation, mediated largely by P2X7R in mouse airways. It has been proposed that P2X7R agonists together with low molecular weight anti-tuberculosis medicines could be used to treat multi-drug-resistant tuberculosis (TB) (Soares-Bezerra et al., 2015), although caution was advised as a polymorphism of the P2X7R was reported to increase the risk of recurrence of TB (Fernando et al., 2007). In infectious inflammatory diseases the roles of P2X7R and ectonucleotidases have been reviewed (Morandini et al., 2014). Polymorphisms of the P2X7R gene are associated with the prognosis and risk of human TB (Zheng X. et al., 2017). Data has been presented to support the view that P2X7R antagonists should be used to treat the aggressive forms of TB (Amaral et al., 2014). Selective, orally bioavailable and potent ATP synthase inhibitors show activity against both non-replicating and replicating TB (Singh et al., 2015). P2X7R contain the spread of *Toxoplasma gondii in vivo* (Corrêa et al., 2017). The role of purinergic signalling in a mouse model of pneumococcal meningitis has been explored (Zierhut et al., 2017). The authors showed that although P2X7R activated the NLRP3 inflammasome/IL-1β pathway that mediates inflammation in pneumococcal meningitis, neither suramin nor brilliant blue G affected the disease, possibly because of meningitis-associated down-regulation of brain P2X7R expression and/or a decrease in ATP levels in cerebrospinal fluid.

Adenosine protects against *Streptococcus pneumoniae* infection of the lungs by pulmonary neutrophil recruitment regulation (Bou Ghanem et al., 2015). Macrophages that engulf bacteria produce adenosine that suppresses sensitisation in response to early-life infections (Pei and Linden, 2016). Chemokine release and leukocyte recruitment are modulated by nucleotides in inflamed airways via an action on P2YR on immune and epithelial cells. Mucociliary clearance is the initial defence against infections of the airways. Airway epithelium releases ATP into the surface liquid layer that controls mucus clearance via P2R and, following breakdown to adenosine, also through P1R. Pulmonary TB patients had higher ADA activity in bronchoalveolar lavage fluid and in the sputum.

Infection with the malaria protozoan parasite, *Plasmodium falciparum*, induces ATP release from erythrocytes. Rupture of erythrocytes releases ATP during the blood-stage of *P. chabaudi* malaria that increases P2X7R expression on CD4^+^ T cells. Platelet ADA, CD39, and CD73 expression was reduced in *Trypanosoma evansi* infected rats. A review about purinergic signalling and malaria-infected erythrocytes is available (Huber, 2012). Haemolysis produced by leukotoxin, a bacterial virulence factor, was increased by ATP release and P2XR activation of human erythrocytes.

P2X7R activation regulates inflammatory responses during acute viral infection (Lee et al., 2012) and is involved in the exacerbated immune response seen during influenza virus infection (Leyva-Grado et al., 2017). ATP, released by activated macrophages and damaged cells, modulates lung inflammation in pneumonia in cattle. Both pulmonary microvascular endothelial cells and epithelial cells expressed P2X7R mRNA.

The pneumovirus respiratory syncytial virus commonly causes childhood lower respiratory tract diseases. It reduces alveolar clearance, probably via UTP, released by the bronchoalveolar epithelium following infection, suggesting that P2Y_2_R antagonists may be therapeutically important for the treatment of severe respiratory syncytial virus bronchiolitis (Vanderstocken et al., 2012). Rhinoviral stimuli and ATP signalling contribute to human bronchial smooth muscle production of IL-33 by severe asthmatics (Calvén et al., 2015). ATP is involved in the expression and release of a major airway mucin, MUC5AC, mainly via P2Y_2_R and it was suggested that modulation of this pathway could be useful clinically for mucus hypersecretion following viral infections (Shishikura et al., 2016).

### Lung Injury

Acute respiratory stress syndrome and lung injury can lead to respiratory failure. There is a protective effect of ATP-MgCl_2_ in ischaemia-reperfusion lung injury. Alveolar macrophages contribute substantially to chronic lung inflammation development, including silicosis, idiopathic pulmonary fibrosis, hypersensitivity pneumonitis, sarcoidosis, and asbestosis.

Alveolar macrophages express P2X7R, which stimulate the IL-1 to IL-5 proinflammatory cytokine cascade and may be clinically relevant in lung hypersensitivity reactions occurring due to chronic inflammation. P2X7R are involved in the pathophysiology of LPS-induced lung injury and LPS-induced inflammation occurs independently of P2Y_1_R (Liverani, 2017). There is up-regulation of pulmonary P2X4 and P2X7R in both acute and chronic lung injury and P2X7R deletion, but not P2X4 deletion, was lung protective (Hafner et al., 2017). The initial inflammatory cells recruited during lung injury are pulmonary neutrophils and P2X7R antagonists reduced neutrophil infiltration and proinflammatory cytokine levels (Mishra et al., 2016).

Neuroendocrine body cells lining the lung epithelium at intervals, release ATP in response to distension, which then stimulates P2X3R to activate vagal sensory fibres that originate in the nodose ganglion (Brouns et al., 2003). This mechanism may control reflex responses to noxious gases and hyperventilation. Ventilator-induced lung injury may involve ATP release from neuroepithelial cell bodies in response to stretch and therefore may be therapeutically relevant.

Pulmonary fibrosis can be caused by injury. In patients with idiopathic pulmonary fibrosis A_2B_R signalling may promote the production of inflammatory and fibrotic mediators. Extracellular adenosine levels are closely associated with the progression of pulmonary fibrosis (Luo F. et al., 2016). Adenosine production by CD73 enhanced radiation-induced lung fibrosis (Wirsdörfer et al., 2016). LPS caused increased expression of A_1_, A_2A_, and A_2B_ and P2YR, and decreased expression of A_3_R, while mechanical ventilation reduced P2Y_4_ mRNA levels. Both A_2A_ and A_2B_R were claimed to attenuate acute lung injury. Upregulated A_2A_R activation is likely to improve the healing process after acute LPS-induced lung injury (Friebe et al., 2014). A protective role for A_2B_R signalling has been reported to counter ischaemic lung injury (Densmore et al., 2017). Protective effects of A_3_R activation in attenuating reperfusion lung injury has also been reported. Inhibition of adenosine kinase attenuates acute lung injury (Köhler et al., 2016). Inhaled A_2B_R agonists have been recommended for the treatment of acute lung injury (Hoegl et al., 2015). Adenosine is detrimental in lung recovery following hyperoxic lung injury (Davies et al., 2016). Reviews have been written about adenosine receptors as potential therapeutic targets for acute respiratory stress syndrome and acute lung injury (Schepp and Reutershan, 2008; Eckle et al., 2009).

### Cystic Fibrosis (CF)

Cystic fibrosis is hereditary as a result of a loss of function gene mutation in the cystic fibrosis transmembrane conductance regulator (CFTR) protein. Some outstanding reviews about purinergic signalling in CF have been published (Burnstock et al., 2012a; Della Latta et al., 2013). The regulation of ion transport by UTP and ATP in CF and normal human airway epithelium was proposed early, in retrospect probably via P2Y_2_ and/or P2Y_4_R. P2R compounds for the treatment of CF to restore Cl^-^ secretion and/or inhibit Na^+^ absorption are being investigated. Lipoxin A_4_, which stops inflammation, is inadequately produced in patients with CF, but it stimulates apical ATP release, which activates P2Y_11_R resulting in epithelial repair (Higgins et al., 2014). With the R117H mutation associated with mild forms of CF, there are faults with the gating conformational changes in the CFTR transmembrane domains, although the function of the nucleotide binding domains are unchanged (Yu et al., 2016). Gating of the R117H-CFTR was shown to be almost completely rectified by combined treatment with an ATP analogue [*N*^6^-(2-phenylethyl)-2′-deoxy-ATP], VX-770 (Ivacaftor, currently used to treat CF) and nitrate ions (NO_3_^-^). The authors concluded that future therapeutic developments might include the complementary use of ATP analogues with VX-770.

Cl^-^ secretion across CF airway epithelia is restored by ATP by triggering calcium entry via P2XR. It was suggested that P2X4/6 heteromultimer receptors might be involved. CFTR activity is necessary for ATP release following erythrocyte deformation. CFTR is reduced or absent in CF and mechanical deformation of erythrocytes does not induce ATP release. Increasing nucleotide release via motion could have therapeutic implications for CF patients. Women with CF showed reduced survival compared with males. Oestrogen may lower breathing-induced release of ATP and ATP receptor-mediated [Ca^2+^]_i_ increase that induces Cl^-^ secretion. Anti-oestrogens may therefore be beneficial in the treatment of CF.

A_1_R agonists release [Ca^2+^]_i_ and activate Cl^-^ and K^+^ currents in CF epithelial cells of the airways. A_1_ and A_2A_R participate in regulation of Cl^-^ secretion in CF airway epithelial cells. Bronchoalveolar lavage from CF patients contained high concentrations of adenosine, correlating with higher 5′-nucleotidase and lower ADA activity. Adenosine regulates CFTR via A_2B_R. The exhaled breath condensate biomarker, adenosine, tracks changes in lung function in CF (Esther et al., 2013). There is increased airway adenosine metabolism in early CF (Esther et al., 2015).

### Lung Cancer

Human lung A549 epithelial-like adenocarcinoma cells express P2UR (i.e., P2Y_2_ and/or P2Y_4_) that mediate increases in [Ca^2+^]_i_. In A549 cells there is calcium-dependent UTP and ATP release (with subsequent increase in adenosine levels). Erythromycin selectively inhibits influx of Ca^2+^ induced via P2X4R stimulation in A549 lung tumour cells. A549 cells express P2Y_2_, P2Y_4_, and P2Y_6_, and P2X4R. ATP induces dose-dependent inhibition of growth of cell lines, including human mesothelioma (MER082), human papillary lung adenocarcinoma (H441), human squamous cell lung carcinoma (H520), human large cell lung carcinoma (H460), human small cell lung carcinoma (GLC4) and the PC14 lung adenocarcinoma cell line. ATP was released from Calu-3 cells derived from human lung adenocarcinoma, probably in response to P2Y_2_R activation. UTP, ATP and uridine diphosphate stimulate proliferation of lung tumour A549 cells through P2Y_2_ and P2Y_6_R.

Autocrine ATP release and P2X7R activation affects the migration of human lung cancer cells (Takai et al., 2014). A significant increase in survival of non-small cell lung cancer patients with high P2X7R expression was identified compared to patients with low P2X7R expression (Boldrini et al., 2015). Nucleotides released by radiochemotherapy induce chemotaxis, adhesion and proliferation of human lung cancer cells and metastasis was inhibited in immunodeficient mice by purinergic receptor antagonists (Schneider et al., 2015). An association of P2X7, P2X4, and P2Y_1_R with distant metastatic lung tumours was observed and increased degradation of ATP and ADP by CD39, which influence tumour growth and metastasisation (Hofman et al., 2015). ATP promotes cell survival by inducing a long lasting and sustained increase in [Ca^2+^]_i_ in lung cancer cells (Song et al., 2016). ATP binding cassette E1 promotes growth, invasion and metastasis of lung adenocarcinoma cells (Tian Y. et al., 2016). Ecto-5′-nucleotidase (CD73) inhibitors are currently under clinical trial to treat non-small cell lung cancer (Zhu et al., 2017).

A_2B_R on host immune cells may participate in promoting angiogenesis and suppressing immunity and A_2B_R KO mice showed reduced growth in a Lewis lung carcinoma isograft model. ATP increased the cytotoxicity of cisplatin, a common anti-cancer drug for the treatment of lung cancer, in the H460 carcinoma cell line. An A_3_R agonist inhibited cell proliferation by stopping the cell cycle and by apoptosis in A549 cells. Antagonism of A_2A_R expressed by lung adenocarcinoma tumour cells inhibited their growth (Mediavilla-Varela et al., 2013). A_2A_R expression and CD73 have opposing prognostic effects in non-small cell lung cancer (Inoue et al., 2017).

Cachexia often occurs in lung cancer patients. In clinical trials, infusion of ATP in advanced non-small cell lung cancer patients contributed beneficially by increasing body weight, muscle strength and quality of life, as well as enhancing survival (Agteresch et al., 2003). ATP has been claimed to reduce radiation-induced damage in patients with non-small cell lung cancer (Swennen et al., 2008).

### Chronic Cough

The therapeutic promise of P2X3R antagonists for the treatment of chronic cough was first recognised by Ford and Undem (2013). P2X3R are expressed by airway afferent nerves and mediate hypersensitivity of the cough reflex, which is dramatically reduced by the oral P2X3 antagonist, AF-219 (Abdulqawi et al., 2015). Central A_1_R were shown to suppress cough (El-Hashim et al., 2016).

### Pleurisy

Inosine contributed with adenosine to exert, via A_2_R, anti-inflammatory effects in pleural inflammation (da Rocha Lapa et al., 2012).

### Lung Allograft

Ecto-5′-nucleotidase (CD73) reduced rejection of airway allografts by stimulating A_2A_R, which is a negative modulator of lymphocyte recruitment into the allograft. P2X7R antagonists prolong mouse lung allograft survival (Liu et al., 2014).

## Diseases of the Special Senses

Purinergic signalling is involved in the physiology of the nasal organs, ear, eye and tongue. Purinergic drugs are being explored for corneal injury, retinal detachment, glaucoma, dry eye, retinitis, uveitis, rhinosinusitis, diabetic retinopathy, macular degeneration, noxious odour damage, Ménière’s disease, sensorineural deafness, tinnitus and taste defects (Burnstock, 2006; Housley et al., 2009).

### Eye

The early literature up to 2006 was reviewed (Burnstock, 2006) and there are more recent reviews covering the treatment of ocular diseases by purinergic drugs (Guzman-Aranguez et al., 2014; Sanderson et al., 2014; Beckel et al., 2016; Lee et al., 2016; Reichenbach and Bringmann, 2016).

Glaucoma is characterised by progressive degeneration of retinal ganglion cells and visual loss. Elevated intraocular pressure reduction is the treatable risk factor for glaucoma. P2X7R antagonists are being explored for the treatment of glaucoma (Krizaj et al., 2014; Pérez de Lara et al., 2015; Sakamoto et al., 2015). There is elevation of extracellular ATP and upregulation of NTPDase1 in animal models of chronic glaucoma (Lu et al., 2015). Cromakalim, a K_ATP_ channel opener, lowers intraocular pressure (Roy Chowdhury et al., 2015, 2016). Evidence for the use of adenosine receptor antagonists for the treatment of glaucoma has also been presented (Zhong et al., 2013; Agarwal and Agarwal, 2014). Ap_4_A improves adrenergic anti-glaucomatous therapeutic effectiveness (Loma et al., 2015) and has recently been claimed to be an effective compound for the treatment of glaucoma (Fonseca et al., 2017).

Treatment for dry eye by a long lasting P2Y_2_R agonist, diquafosol, was developed by Inspire Pharmaceuticals, Inc. and is currently in use in Japan and Korea (see Lau et al., 2014).

A P2Y_2_R agonist, INS37217, enhances subretinal fluid reabsorption and is recommended for the treatment of retinal detachment (Maminishkis et al., 2002; Meyer et al., 2002).

P2X7R antagonist and A_3_R agonists have been implicated for the treatment of diabetic neuropathy and retinopathy (Sugiyama, 2014; Reichenbach and Bringmann, 2016), photoreceptor neurodegeneration (Hu et al., 2015; Ho T. et al., 2016), retinitis (Corso et al., 2016) and uveitis (Zhao R. et al., 2016).

The roles of P2Y_2_ and P2X7R in corneal wound healing have been reviewed (Minns and Trinkaus-Randall, 2016; Minns et al., 2016). P2X7R activation in oxysterol cytotoxicity may be a target for the treatment of age-related macular degeneration (Olivier et al., 2016).

### Ear

In the auditory system ATP depressed sound-evoked action potentials in the auditory nerve by stimulating P2YR. Acoustic over-stimulation can cause permanent hearing loss due to damage and death of cochlea hair cells. Noise exposure promotes the release of ATP into endolymph. ATP regulates hearing sensitivity and could be useful to treat sensorineural deafness, Ménières disease and tinnitus. UTP infusion into the deafened guinea pig inner ear rescued auditory neurons (Fransson et al., 2009). P2XR-mediated control of cochlear gap junctions may be protective by reducing hearing sensitivity to noise stress (Zhu and Zhao, 2012), perhaps via P2X2R (Yan et al., 2013; Mittal et al., 2016). Susceptibility to hearing loss and noise-induced neural injury in the mouse cochlea is regulated by A_2A_R (Vlajkovic et al., 2017).

### Olfactory Organs

Purinergic receptors are expressed in the nasal mucosa, including P2X3R on olfactory neurons. There is enhanced odour sensitivity in the presence of antagonists to P2R suggesting that endogenous ATP at a low level normally decreases odour responsiveness. Chemosensory trigeminal neurons express P2X2R, which contribute to control of odour recognition (Housley et al., 2009). Activation of P2X3R negatively modulates the odour response, indicating a protective strategy for olfactory sensory neurons (Yu, 2015). Olfactory nerves and secretory cells in the vomeronasal organ express purinoceptors. Heat-shock protein induction by noxious odour damage is inhibited by P2R antagonists *in vivo*. The inhibitory effect of ATP in odour responses may contribute to the reduction of odour sensitivity following exposure to noxious fumes and could be a new mechanism for neuroprotection (Yu and Zhang, 2014). ATP release following injury leads to post-injury neuroregeneration and may result in the development of therapies to restore loss of smell (Hayoz et al., 2012). P2Y_1_R increase neuronal network activity in the developing olfactory bulb (Fischer et al., 2012). Purinergic signalling serves as a paracrine signal in regulating the neurogenesis of mouse olfactory epithelium (Gao L. et al., 2010). It has been claimed that purinoceptors are a therapeutic target to alleviate or restore loss of olfactory sensory neurons by the mycotoxin, satratoxin (Jia et al., 2011). Activation of A_2_R may be a novel therapeutic approach for enhancing nasal mucociliary clearance in chronic rhinosinusitis (Hua et al., 2013).

### Tongue

ATP is a key neurotransmitter in the taste system, acting largely via P2X2/3 heteromultimer receptors (Kinnamon and Finger, 2013; Vandenbeuch et al., 2015). Consequently, disruption of taste function may be an unintentional consequence of therapeutic trials of pain, chronic cough and other conditions using purinergic P2X3R antagonists (Vandenbeuch et al., 2013).

## Immune System and Inflammation

P2X7, P2Y_1_, and P2Y_2_R on immune and inflammatory cells are important in immunomodulation and inflammation, and the purinergic contribution to neuroinflammation underlying neuropathology has been discussed in several recent reviews (Jacob et al., 2013; Takenouchi et al., 2014; Di Virgilio and Vuerich, 2015; Beamer et al., 2016). The role of P2X7R in particular in diseases related to neuroinflammation and the use of P2X7R centrally penetrant antagonists has been highlighted (Baudelet et al., 2015; Di Virgilio, 2015; Gentile et al., 2015; Burnstock, 2016b; Danquah et al., 2016; Karmakar et al., 2016; Rech et al., 2016; Corrêa et al., 2017; Giuliani A.L. et al., 2017; Jiang et al., 2017; Zheng B. et al., 2017). It has been claimed that activation of P2X1R on neutrophils and platelets is involved in regulation of thrombo-inflammation (Oury et al., 2015). P2Y_12_R activation modulates sepsis-induced inflammation (Liverani et al., 2016). Adenosine, acting mainly via A_2A_R, is also involved in neoplastic and inflammatory and immune-mediated disease states (Antonioli et al., 2014a; da Rocha Lapa et al., 2014; Cekic and Linden, 2016; Ingwersen et al., 2016; Zhang X. et al., 2016; Faas et al., 2017).

## Infection

Reviews have been published concerned with purinergic signalling in immune cell trafficking at sites of infection (Ferrari et al., 2016b) and P2X7R in infectious inflammatory diseases (Morandini et al., 2014). Apoptosis of macrophages via ATP-stimulated P2X7R leads to the killing of the mycobacteria they contain, which may lead to new strategies to combat bacterial infections. The cytotoxic effects of ATP on macrophages are through P2X7R, while the bactericidal actions of ATP (and UTP) may be though P2Y_2_R. There is a valuable article about purinergic signalling in infection and autoimmune diseases (Savio and Coutinho-Silva, 2016). The P2X7R is a potential target for the treatment of *Clostridium perfringens* type C infection (Nagahama et al., 2015). P2X7R activation modulates cell death during *Porphyromonas gingivalis* infection (Almeida-da-Silva et al., 2016). P2X7 and P2X4R activation is protective during severe *Escherichia coli* infection (Greve et al., 2017). P2X7R develop the inflammatory response associated with sepsis (Santana et al., 2015) and might serve as a therapeutic target to ameliorate brain damage in sepsis (Savio et al., 2016). A_2A_R have also been recommended as a therapeutic target to treat sepsis (Sivak et al., 2016). The role of purinergic signalling in the immune response in sepsis has been reviewed (Ledderose et al., 2016). ATP protects against sepsis through P2X7R on macrophages by enhancing intracellular bacterial killing (Csóka et al., 2015). *Chlamydiae* are intracellular bacterial pathogens and these infections are influenced by inflammasomes and purinergic signalling (Pettengill et al., 2016). Purines modulate the inflammatory response in rats infected by *Cryptococcus neoformans* (de Azevedo et al., 2016). ADP facilitates monocyte recruitment in bacterial infection (Zhang X. et al., 2016). ATP synthase has been proposed as a target to kill *Mycobacterium tuberculosis* (Tantry et al., 2017). Purinergic enzymatic activities in lymphocytes and cardiomyocytes modulated the inflammatory responses of mice infected by *T. cruzi* (do Carmo et al., 2017). Adenosine restored LPS-inhibited chemotaxis via A_1_R, making this a promising therapeutic strategy for infectious diseases (Xu et al., 2017).

Infection by *Schistosoma mansoni*, a parasitic blood fluke, results in thymic atrophy. The cloning and characterisation of a P2XR (schP2X) from *S. mansoni* was the first non-vertebrate ATP-gated ion channel, which could be an alternative drug target to treat schistosomiasis (see Burnstock and Kennedy, 2011). Purinergic signalling influences the immune response to infection by *Leishmania*, a protozoan parasite (Chaves et al., 2014; Figueiredo et al., 2016). Purinergic signalling is involved in *Trichomonas vaginalis* parasite infection (Menezes and Tasca, 2016). The dysfunction of P1, P2Y and P2X7R and NTPDases are likely to contribute to morbidity due to human schistosomiasis (Silva, 2016). P2X7R are important in parasite control as they regulate T effector cells and inflammation during *L. amazonensis* infection (Figliuolo et al., 2017a). A commentary about ATP as an initiator of immunity to parasitic infections has been published (Nelson et al., 2017).

P2X7R modulate the antiviral and inflammatory processes that occur during Dengue virus-2 infection (Corrêa et al., 2016) and exacerbate the immune response that occurs during influenza virus infection (Leyva-Grado et al., 2017). P2X4R antagonists reduce herpetic pain (Matsumura et al., 2016). Both P2X4 and P2X7R are involved in hepatitis C virus infection (Manzoor et al., 2016). Purinoceptors are putative targets for the treatment of HIV-1 infection (Pacheco et al., 2014). Ribavirin, an adenosine analogue, exhibited potent antiviral activities (Hao et al., 2017).

## Endocrine Diseases

Purinoceptors are expressed widely by endocrine glands (Burnstock and Knight, 2004). Actions of purines have been described in the pituitary gland, with implications for pathological as well as physiological states. 5′-Nucleotidase activity in platelets is changed by hyper- and hypothyroidism and could be a further mechanism by which alterations in thyroid hormones are related to vascular diseases. The role of purinergic signalling in thyroid hormone activities in health and disease has been reviewed (Silveira et al., 2013). In the ovary, follicular atresia involves cellular degeneration that is due to apoptosis evoked by P2X7R activation in both somatic and germinal follicular cells. Adiponectin, secreted by adipocytes, is anti-inflammatory, protecting against fatty liver disorder, insulin-resistant type 2 diabetes and atherosclerosis. A review including the limited knowledge of purinergic signalling in the endocrine system has been published (Burnstock, 2014b).

### Diabetes

Fibroblast P2YR are impaired in type 2 diabetes, which leads to lower glucose uptake, indicating that P2YR could be therapeutically useful as antidiabetic drugs. Analogues of the P2Y_1_R agonist, 2-methylthio ADP, have been developed for the treatment of type 2 diabetes (Yelovitch et al., 2012). P2YR activation potentiates insulin secretion making it a promising therapeutic target for type 2 diabetes (Zhang et al., 2015b; DeOliveira et al., 2017). Adenosine, acting via A_2A_R, increases pancreatic β-cell proliferation, and has been suggested as a therapeutic target for diabetes (Schulz et al., 2016). Uridine adenosine tetraphosphate may be a therapeutic target for diabetes (Matsumoto et al., 2015).

There is an enhancement of P2X7R-induced apoptosis on the retinal microvasculature in early diabetes. P2X7R located on glucagon-containing α cells in pancreatic islets in STZ-induced diabetic animals increase and they migrate centrally to replace missing insulin-containing β cells. P2X7R antagonists have been proposed as a therapeutic target to cause immunosuppression and tolerance induction in pancreatic islet transplantation (Aikin, 2013; Vergani et al., 2013). NONRATT021972 siRNA decreases the expression of P2X7 mRNA and protein in DRG, reducing mechanical and thermal hyperalgesia in type 2 diabetic rats (Liu et al., 2016c). Type 1 diabetes impairs P2X7R signalling in osteocytes that affects osteoblast function and maintenance of bone health (Seref-Ferlengez et al., 2016). STZ-induced type 1 diabetes was prevented in P2X7R KO mice (Vieira et al., 2016). Diabetic sympathetic neuropathy in type 2 diabetic rats was reduced by decreasing the expression of P2X7R in superior cervical ganglia (Wu et al., 2016). P2X7R are expressed in pancreatic cancer cells. P2X7R antagonists are likely to be effective therapeutic agents (Giannuzzo et al., 2016).

P2X3R antagonists have been proposed for the treatment of diabetic neuropathic pain (Guo et al., 2015; Zhang et al., 2015b; Peng et al., 2017; Rao et al., 2017). Recently P2X7R have also been claimed to be involved in painful diabetic neuropathy in rats (Liu et al., 2017). P2X7R polymorphisms are associated with severe diabetic neuropathic pain scores (Ursu et al., 2014). A_1_R agonists improve mechanical allodynia in a painful diabetic neuropathy mouse model (Katz et al., 2015).

There is up-regulation of hippocampal A_2A_R in STZ-diabetes and A_2A_R antagonists gave neuroprotection. A_2A_R are a therapeutic target for diabetic retinopathy (Ibrahim et al., 2011). Reduced expression of A_1_R in pancreatic α-cells could result in the development of type 1 diabetes (Yip et al., 2013). There is increased expression of A_2B_R in women with gestational diabetes mellitus (Wojcik et al., 2014). In diabetic rats, A_2B_R agonists improve erectile function (Wen et al., 2015). It was suggested that reduction in A_3_R expression/function may slow the progression of diabetic neuropathy (Yan H. et al., 2016). Blockade of ATP synthase interaction with cyclophilin D has been proposed as a promising therapeutic target to treat diabetic encephalopathy (Yan S. et al., 2016).

K_ATP_ channels have neuroprotective effects in patients with type 2 diabetes (Liu R. et al., 2016; Rubaiy, 2016). P2Y_1_ and P2Y_2_R mediate relaxation of the rat corpus cavernosum and may improve erectile function in men with diabetes (Gür et al., 2009).

An A_2A_R agonist was shown to enhance healing of chronic diabetic foot ulcers in a clinical trial (Squadrito et al., 2014; Montesinos et al., 2015). Adenosine kinase inhibitors attenuate inflammation in diabetic retinopathy (Elsherbiny et al., 2013a). Retinal inflammation in diabetic retinopathy is mediated by ADA2 and the anti-inflammatory activity of A_2A_R signalling is impaired with increased ADA2 activity (Elsherbiny et al., 2013b).

Several reviews about purinergic signalling in diabetes and its therapeutic potential are available (Burnstock and Novak, 2013; Cieslak and Roszek, 2014; Antonioli et al., 2015; Fotino et al., 2015; Kishore et al., 2015; Merighi et al., 2015; Vindeirinho et al., 2016). The involvement of purinergic signalling in diabetic nephropathy is discussed later in the Section on Kidney.

## Obesity

ATP released as a cotransmitter from sympathetic nerves, stimulates brown adipocytes. ATP stimulates lipogenesis in rat adipocytes, regulating fat stores not via established hormones. Adipocytes express two different P2YR subtypes and P2Y_11_R activation inhibited insulin-stimulated lepton production and lipolysis stimulation. P2Y_4_R mediate inhibition of cardiac fat formation (Lemaire et al., 2016). The anti-obesity effects of sesamol, a potent anti-inflammatory compound, is mediated by AMP-activated protein kinase (Go et al., 2017). Blocking P2Y_6_R activation in the CNS with the antagonist MRS2578 inhibits feeding in mice (Steculorum et al., 2017).

Some ATP effects are as a result of adenosine, after breakdown of ATP, which is also involved in the activities of adipocytes. Adenosine increased lipolysis and induced thermogenesis in brown adipocytes via A_2A_R (Gnad et al., 2014). They showed that A_2A_R antagonists counteract high fat-induced obesity in mice. Administration of A_2A_R agonists to obese mice caused improvements in glucose homeostasis and adipose tissue inflammation, suggesting that this may show promise for therapeutic treatment of obesity (DeOliveira et al., 2017).

There is disturbance of adiponectin secretion in obese patients and adiponectin release is controlled by ATP (Yamauchi and Kadowaki, 2013). A combination of Ca^2+^ and ATP augments vesicular release of adiponectin (Komai et al., 2014). Both ATP and adenosine have roles in the regulation of leptin secretion from adipocytes (Szkudelski, 2007). There is abnormal fat distribution in P2X7R KO mice (Beaucage et al., 2014). Manipulation of P2XR signalling may represent a novel therapeutic target in metabolically unhealthy obesity under inflammatory conditions (Pandolfi et al., 2016). There is protection of rats on high fat diet by adenosine (Lee, 2015). Perivascular adipose tissue of heavy smokers has increased expression of P2X7R and inflammasome components leading to increased release of inflammatory cytokines (Rossi et al., 2014).

## Gut Disorders

Purinergic signalling plays an important role in a variety of gut activities (Liñán-Rico et al., 2015; Burnstock, 2016d; Chaudhury et al., 2016). ATP is a cotransmitter in non-adrenergic, non-cholinergic inhibitory nerves involved in peristalsis, a synaptic transmitter in submucosal and myenteric ganglia, it mediates mucosal secretion and vascular control of the gastrointestinal tract. Both interstitial cells of Cajal and enteric glial cells express P2R.

Investigations of purinoceptors as therapeutic candidates for gut disorders are underway (Antonioli et al., 2013; Ochoa-Cortes et al., 2014; Burnstock, 2016d).

### Inflammatory Bowel Disease (IBD)

Nucleotides and their receptors are involved in the pathogenesis of IBD, of which ulcerative colitis (UC) and Crohn’s disease are the main types. P2Y_6_R are highly expressed on the T cells infiltrating IBD, suggesting that P2Y_6_R may play a role in the pathogenesis of IBD. P2X7R agonists are involved in colonic motor dysfunction associated with bowel inflammation in rats (Antonioli et al., 2014b) and are over-expressed in gut mucosa of patients with IBD. P2X7R KO mice are protected against gut inflammation (Figliuolo et al., 2017b). ATP activates mast cells, which further promote the inflammatory process (Kurashima and Kiyono, 2016). Oestrogen receptor β activation plays a therapeutic role in IBD by down-regulation of P2X3 and P2X7R (Ma B. et al., 2016). The roles of P2X7R in IBD are discussed in reviews (Kurashima et al., 2015; Diezmos et al., 2016).

P2X7R-expressing enteric neurons are differentially affected in UC based on their chemical codes (da Silva et al., 2015). In a later paper, it was shown that UC affected secretory and vasodilatory neurons, enteric sensory neurons and enteric glia of the submucosal plexus expressing P2X7R (da Silva et al., 2017). The P2X7R antagonist, A438079, down-regulated the production of proinflammatory cytokines and attenuated murine colitis, indicating that P2X7R mediate inflammatory responses during UC (Wan P. et al., 2016). Activation of P2X7R triggers the death of mucosal regulatory T cells (Figliuolo et al., 2017b). There was P2XR enhancement in an animal model of UC. MicroRNA-16 and microRNA-206 have pro-inflammatory roles in UC by down-regulating A_2A_ and A_3_R expression (Tian T. et al., 2016; Wu et al., 2017).

There is increased expression of P2X7R in the inflamed mucosa in Crohn’s disease, suggesting that P2X7R antagonists may be targets for treatment of Crohn’s disease (Neves et al., 2014; Eser et al., 2015; Wan P. et al., 2016; Figliuolo et al., 2017b). P2X3R antagonists are being explored as therapeutic agents against colic and UC pain (Eser et al., 2015), as well as antibodies against P2X3R (Deiteren et al., 2015; Diezmos et al., 2016; Shcherbatko et al., 2016).

The potential of P2X3R to treat irritable bowel syndrome has been suggested. In diarrhoea-predominant irritable bowel syndrome there is increased expression of P2Y_1_ and P2Y_2_R, which was associated with abdominal pain (Luo Y. et al., 2016). P2X7R in DRG play a role in transmission of the nociceptive signal from the gut (Liu S. et al., 2015). A review about purinergic mediators in the control of intestinal inflammation and irritable bowel syndrome is available (Kurashima et al., 2015).

### Motility Disorders

Purinergic signalling is involved in gastrointestinal motility disorders, including diarrhoea and constipation (Jiménez et al., 2014). Purinergic fast inhibitory junction potentials are impaired in Hirschsprung’s disease (Jiménez et al., 2015). P2X4 and P2X7R activity was potentiated in enteric glia isolated from mice treated long-term with morphine (Bhave et al., 2017).

A review focuses on the pathophysiological roles of P2YR in inflammation (Wan H.X. et al., 2016). Aged mice have a lower ability to deal with inflammation evoked by *Candida albicans* infection, due to a lower gut density of A_2A_R, which reduce inflammation (Rodrigues et al., 2016).

### Colorectal Cancer

Apoptosis is induced by extracellular ATP and ATP also reduced growth of primary cultures of colorectal carcinomas, possibly via P2Y_2_R (see Burnstock and Di Virgilio, 2013; Wan H.X. et al., 2016). Purinergic responses of HT-29 cells, a colonic adenocarcinoma cell line, are mediated by P2Y_2_ and/or P2Y_4_R. Enhanced expression of A_2B_R on colorectal cancer cells that are proliferating suggests that antagonists to A_2B_R may be a promising therapeutic target for colorectal cancer (Ma et al., 2010; Molck et al., 2016). 8-Chloro adenosine inhibited growth of colorectal cancer cell lines 80514 and HCT116 *in vivo* and *in vitro*. A phase II, multi-centre study of CF101, an A_3_R agonist, showed stabilisation of tumour in 35% of refractory metastatic colorectal cancer patients. P2X7R antagonists reduce tumour occurrence in a colitis-associated cancer mouse model (Hofman et al., 2015). There is high expression of the ectonucleotidase CD39 in human rectal adenocarcinoma (Zhang et al., 2015a).

## Diseases of the Kidney

Reviews are available (Burnstock et al., 2014a; Menzies et al., 2017; Oyarzun et al., 2017), including one that focuses on the roles played by ATP released as a cotransmitter from sympathetic nerves in renal diseases (Burnstock and Loesch, 2017).

Purinoceptors are richly expressed in the glomerulus, renal vascular system and nephron in the kidney, including subtypes involved in the regulation of glomerular filtration, renin secretion and transport of nutrients, ions, water and toxins. The distribution of NTPDases 1 and 2 parallels the distribution of P2R in the kidney, influencing physiological and pathophysiological renal events (see Burnstock et al., 2014a). Adenosine and ATP have protective effects against renal ischaemic-reperfusion injury, and have been investigated for transplantation-induced erythrocytosis and chronic renal failure treatment. It has been suggested that nephron luminal P2R are part of an epithelial ‘secretory’ defence mechanism against harmful particles or bacteria. Increased expression of P2X7R in glomerulonephritis, hypertension, polycystic kidney disease (PKD) and diabetes may be therapeutically relevant as a novel treatment of kidney failure.

### Renal Injury and Failure

ATP contributes to kidney remodelling and progression toward chronic renal failure (with associated sympathetic overactivity). P2X7R are expressed weakly in healthy glomerulus, but after glomerular injury (e.g., in hypertension and diabetes) are upregulated, mostly in podocytes, but also in mesangial and endothelial cells (Vonend et al., 2004). P2X7R play a role in altered intracellular calcium homeostasis in peripheral blood mononuclear cells of chronic kidney disease patients (Lajdova et al., 2012). P2X7R mediate deleterious renal epithelial-fibroblast cross-talk. P2X7R antagonists may be therapeutic targets to prevent and treat morbidity and mortality in kidney injury (Zarjou and Agarwal, 2011). P2X7R antagonists were protective against ischaemic acute kidney injury in mice (Yan et al., 2015). Arterial calcification is a feature in chronic kidney disease patients and ATP signalling is involved (see Fish et al., 2013). Cyclosporine is a potent immunosuppressive agent, but with limitations because of its side effect of nephrotoxicity. However, treatment with ATP following pre-treatment with verapamil greatly reduces nephrotoxicity. ATP is an important contributor to innate immunity regulation in primary idiopathic nephrotic syndrome. There is increased renal fibrosis in P2X4R-deficient mice following unilateral ureteric obstruction (Kim et al., 2014).

Adenosine mediates haemodynamic changes in adult renal failure. A_2B_R-mediated IL-6 induction contributes to renal fibrogenesis and this receptor has therapeutic potential for treatment of chronic kidney disease (Grenz et al., 2012). Dendritic cells and macrophages activated by A_2A_R agonists attenuate acute renal injury (Li et al., 2012; Truong et al., 2016). There is a review describing A_2A_R in acute kidney injury (Vincent and Okusa, 2015).

### Polycystic Kidney Disease (PKD)

The genetic disorder PKD results in abnormal proliferation of tubular cells of the adult nephron, leading to progressive dilation of tubules and formation of fluid-filled cysts that destroy by compression neighbouring tissue. In the cysts ATP is released at high concentrations. The *Caenorhabditis elegans* nematode is an animal model for investigating basic molecular mechanisms underlying human autosomal dominant PKD; the *C. elegans* PKD-2 and LOV-1 proteins are homologues of human polycystin (PC)-1 and PC-2 proteins. Nucleotide release is involved in both fluid flow and pressure responses and its role in altered mechanosensory transduction in PKD is considered in a review (Patel and Honoré, 2010).

Cysts in the collecting ducts of the *cpk/cpk* mouse model of congenital PKD express P2X7R, where they mediate cyst development. P2Y_1_, P2Y_2_, P2Y_4_, P2Y_6_, P2X5, and P2X7R were found on the lining epithelial cells of renal cysts in the rat Han:SPRD cy/+ model of autosomal dominant PKD. Expression of mRNA and protein for P2Y_2_, P2Y_6_, and P2X7R increased substantially as the disease developed. ATP inhibits renal cyst growth, via P2X7R. The P2X7R antagonists, oxidised ATP and A-438079, reduced cyst formation via extracellular signal-regulated kinase-dependent pathways in a zebrafish model of PKD (Chang et al., 2011). Nucleotides in the lumen fluid of cysts stimulate P2YR resulting in increased growth of Madin Darby canine kidney-derived cysts. Knocking out PKD-1 gene expression increased A_3_R in human renal cells (Aguiari et al., 2009). There is a review about the functional and therapeutic importance of purinergic signalling in PKD (Ilatovskaya et al., 2016).

### Ischaemia

Proximal tubular ATP declines rapidly in ischaemic acute renal failure. There was early recognition that there was restoration after ischaemia by perfusion of ATP-MgCl_2_ and A_3_R KO mice were shown to be protected against ischaemic renal failure. The ectonucleotidase CD73 protects the kidney from ischaemia-reperfusion injury via production of adenosine and free radical reduction (Jian et al., 2012). The role of adenosine in protection from renal ischaemia-reperfusion injury has been investigated and discussed in reviews (Yap and Lee, 2012; Roberts et al., 2013; Sashindranath et al., 2017).

### Nephritis

Glomerulonephritis is one of the leading causes of end-stage renal disease. Increased expression of pro-apoptotic P2X7R has been demonstrated in both experimental and human glomerulonephritis, which suggests that P2X7R antagonists may have therapeutic potential (Deplano et al., 2013). It was shown later that P2X7R-deficiency attenuated renal injury in experimental glomerulonephritis (Taylor et al., 2009). P2X7R blockade attenuates lupus nephritis by inhibiting inflammasome activation (Zhao et al., 2013) and protects against cisplatin-induced nephrotoxicity (Zhang et al., 2014). The P2R antagonist, pyridoxal-phosphate-6-azophenyl-2′,4′-disulfonate (PPADS), effectively inhibits mesangial cell proliferation in a rat mesangial proliferative glomerulonephritis model. Tubuloglomerular feedback is greatly reduced in Thy-1 nephritic rats, but exogenous 5′-nucleotidase improved it. In P2Y_1_R KO mice, there is protection against fibrosis and death by renal failure as a result of experimental crescentic glomerulonephritis. Adenosine uptake inhibitors attenuated glomerulonephritis in mice. A_2A_R agonists may treat macrophage-mediated experimental glomerulonephritis. A_2A_R agonists reduced inflammation in mouse kidneys, suggesting a therapeutic approach for human lupus nephritis.

### Hypertension

Hypertension is a feature of chronic renal disease; this is due largely to sympathetic overactivity as a result of afferent signals from the kidney and triggering sympathetic tone resetting by activation of hypothalamic centres (Orth et al., 2001). ATP is a cotransmitter released from sympathetic nerves, whose activity is increased in hypertension.

There is an enhanced P2R-mediated vasoconstriction of both efferent and afferent arterioles in chronic angiotensin II-induced hypertensive rats, with predominant P2X1 and P2X7R control of glomerular haemodynamics (Franco et al., 2017). In connexin 30 KO mice, epithelial sodium channels that are unable to respond to changes in sodium levels as a result of reduced paracrine ATP feedback regulation may play a role in salt-sensitive hypertension. Mice lacking P2Y_2_R have salt-resistant hypertension. The P2X7R is expressed weakly in healthy kidney glomerulus, but expression is significantly increased in hypertension (Vonend et al., 2004; Menzies et al., 2015). P2X7R antagonists inhibited the development of renal injury and salt-sensitive hypertension in Dahl salt-sensitive rats (Ji et al., 2012). Activation of A_1_R in mouse proximal tubules, modulated deoxycorticosterone acetate-salt hypertension.

### Diabetic Nephropathy

Adenosine receptor agonists protect STZ-diabetic rats from nephropathy (Taskiran et al., 2016). A review discusses the role of A_2B_R antagonists as therapeutic treatment of diabetic nephropathy (Quezada et al., 2013). CD73 generation of adenosine attenuates diabetic nephropathy (Tak et al., 2014). A_3_R antagonists reduced fibrosis in diabetic nephropathy (Kretschmar et al., 2016). AMP-activated protein kinase has been proposed as a potential treatment of diabetic nephropathy (Cameron et al., 2016). Chronic A_2A_R stimulation prevents proteinuria and glomerular damage in experimental diabetes (Persson et al., 2015).

P2X7R agonists evoke renal inflammation and injury induced by high-fat diet in type 2 diabetes, suggesting that P2X7R antagonists might be of therapeutic interest (Solini et al., 2013). P2X7R expression was increased in the STZ diabetic rat model (Vonend et al., 2004). It has been suggested that P2X7R antagonists may be a useful coadjuvant treatment to delay the progression of diabetic nephropathy (Rodrigues et al., 2014). Data has been presented to suggest that activation of P2X7R contributes to the high prevalence of kidney disease found in diabetics (Rodrigues et al., 2014; Menzies et al., 2017). Deletion of the P2Y_2_R reduced the development of nephrogenic diabetes insipidus polyuria induced by lithium. Blockade of P2Y_12_R in the renal collecting duct alleviates nephrogenic diabetes insipidus (Zhang et al., 2015c).

### Nephrotoxicant Injury

Drug-induced nephrotoxicity was reduced by ATP-MgCl_2_. A_1_R antagonists are protective against cisplatin-induced acute kidney injury in rats (Gill et al., 2009). Adenosine antagonists are protective against acute renal failure. A_1_R antagonists also reduce nephrotoxicity induced by the immunosuppressive agent cyclosporine. Cyclosporine increases plasma adenosine levels in kidney transplant patients. A_3_R antagonism is effective against acute tacrolimus toxicity.

### Cancer

P2X7R are strongly expressed on human embryonic kidney tumours, associated with increased proliferation (Adinolfi et al., 2012). The anthraquinone, emodin, inhibits human embryonic kidney cancer cell invasiveness by antagonising P2X7R (Jelassi et al., 2013). A prognostic indicator for post-operative cancer-specific survival of clear-cell renal cell carcinoma patients is P2X7R expression (Liu Z. et al., 2015).

## Diseases of the Lower Urinary Tract

The early literature has been thoroughly reviewed (Burnstock, 2014d; Keay et al., 2014).

### Urinary Bladder

In contrast to laboratory animals, where the purinergic component of bladder parasympathetic cotransmission is about 50%, in healthy human bladder it is approximately 2% although ATP receptors are expressed by the smooth muscle. In pathological conditions, however, such as neurogenic bladder, outflow obstruction and interstitial cystitis (IC), the purinergic component can be as much as 40% and is consequently a therapeutic target. Botulinum neurotoxin type A (BTXA) is often used to treat bladder incontinence, where it inhibits ATP release as well as acetylcholine (ACh) release both from parasympathetic nerves and urothelial cells. Bladder epithelial cell release of ATP from patients with IC was substantially higher compared to healthy controls. Dysfunction in micturition involves P2X3R in rats with chronic spinal cord-injury, suggesting that P2X3R antagonists could be used to treat neurogenic bladder dysfunction.

#### Overactive Bladder (OAB) Syndrome

There are valuable reviews that discuss the roles of purinergic signalling in OAB (Meng et al., 2012; Sacco et al., 2015). OAB syndrome, which increases in old age, is characterised by frequency, nocturia and urgency, with or without urge incontinence. Women with an OAB exhibit higher ATP levels in their urine compared to controls, suggesting that this could be used as a prognostic marker for detrusor overactivity (Cheng et al., 2013; Silva-Ramos et al., 2013). The increased urine ATP concentration is due to increased release of ATP from proliferating urothelium and reduced metabolism of ATP (Silva-Ramos et al., 2013). P2X3R are present in the human bladder and, when upregulated, may play a role in the pathophysiology of OAB.

Patients with metabolic syndrome, where the risk of developing cardiovascular disease and diabetes is increased, exhibit a higher incidence of OAB. P2X3R expression is increased in subepithelial sensory nerves resulting in increased bladder activity. OAB is common among PD patients and inhibition of overactivity by A_2A_R antagonists occurs, probably by acting in the CNS to regulate the micturition reflex (Kitta et al., 2012). Uridine diphosphate via P2Y_6_R regulates abnormal smooth muscle activity in the OAB and increases contractions due to P2X1R activation (Yu W. et al., 2013). P2Y_6_-deficiency increases micturition frequency and reduces contractility in the mouse urinary bladder (Kira et al., 2017).

#### Unstable Bladder

Unstable bladder (also referred to as detrusor instability or detrusor hyperreflexia) is either neurogenic or idiopathic in origin. Neurogenic detrusor instability can occur as a result of a stroke, or pelvic or spinal cord injury, as well as in PD and MS. Neurogenic bladders are hyper-responsive to ATP, via P2X3R.

Expression of P2X2R in both urothelium and detrusor muscle of suprasacral spinal cord injury patients are similar to P2X2R expression in bladder tissue from idiopathic overactive bladder patients. ATP levels increased in the rat spinal cord-injured bladder that activates P2X3 and P2X2/3R on afferent nerve fibres resulting in neurogenic bladder overactivity (Munoz et al., 2012). P2YR agonists ADP, UTP and uridine diphosphate originating in the urothelium augment the contractile overactivity following spinal cord injury. Sympathetic nerve hyperactivity may play a role in reflex sympathetic dystrophy where increased ATP would be released as a cotransmitter to activate P2X1R on smooth muscle resulting in increased contractions of the bladder and P2X3R on sensory nerve fibre terminals resulting in bladder reflexes and nociception. Inhibition of P2X7R expressed at spinal cord injury sites improved the dysfunction of neurogenic bladder (Munoz et al., 2017).

Urothelial cell ATP release (both resting and evoked) was substantially more from rat bladders following chronic spinal cord injury, contributing to bladder hyperactivity development. In a mouse bladder overactivity model, bradykinin facilitated the release of ATP from nerve terminals via prejunctional receptors (Fabiyi and Brading, 2006).

In the absence of P2X3R in KO mice, there is bladder hyperactivity (Cockayne et al., 2000; Vlaskovska et al., 2001). AF-219, a P2X3 and P2X2/3R antagonist that is metabolically stable and orally bioavailable, is being investigated as a treatment for urinary tract dysfunction (Ford and Cockayne, 2011).

Idiopathic detrusor instability patients show abnormal bladder purinergic transmission, which may contribute to the symptoms of OAB (Andersson and Hedlund, 2002; O’Reilly et al., 2002). There is greater potency of ATP via P2X1R for generating detrusor contractions in unstable bladder patients. Urothelial release of ATP from human bladders from patients with detrusor overactivity occurs in both idiopathic and neurogenic conditions (Kumar et al., 2010). Oxybutynin, administered chronically, results in a shift from muscarinic to purinergic transmission in the rat bladder wall. Expression of P2X3R is increased on suburothelial sensory nerve fibres in idiopathic detrusor overactivity patients (Liu H. et al., 2013).

#### Interstitial Cystitis (IC)/Bladder Pain Syndrome

The possible roles of ATP and adenosine in IC/bladder pain syndrome are discussed in a review (Veselá et al., 2012a). Hypoosmotic mechanical stimulation of urothelial cells released ATP that was substantially increased in cyclophosphamide-induced inflamed bladder (Smith et al., 2005). In rats with cystitis induced by hydrochloric acid, purinergic receptors were lost, although release of ATP from mucosal cells was increased.

Contractions of the rat bladder mediated by parasympathetic nerve stimulation and release of ATP and ACh, were reduced in cyclophosphamide-induced cystitis (Veselá et al., 2012b), while P2XR function in sensory neurons was enhanced. The purinergic component was abolished in the neurogenic bladder following desensitisation with α,β-methylene ATP (α,β-meATP). ATP release due to stretch from bladder urothelium from IC patients was greater compared to healthy urothelium and P2X3R expression was upregulated. Urothelial cells from cats with feline IC responded to α,β-meATP, indicating increased expression of urothelial P2XR. There is mechanical hypersensitivity of the bladder in feline IC together with greater release of ATP from the urothelium; P2X1R expression is reduced and P2Y_2_R expression is lost.

A_1_R blockade during the initial phase of IC/bladder pain syndrome was suggested as a treatment for this condition (*Aronsson et al., 2012*). The A_1_R in rat urinary bladder is decreased in cyclophosphamide-induced cystitis (Veselá et al., 2011).

In neutrophils and macrophages in the submucosa of the bladder from mice with cyclophosphamide-induced haemorrhagic cystitis, expression of P2X7R is increased (Martins et al., 2012). Treatment with A-438079, a P2X7R antagonist, or genetic ablation of this receptor reduced nociceptive behaviour.

#### Outflow Obstruction

P2X1R expression in smooth muscle of the bladder was increased in the human obstructed bladder. In animal models of outlet obstruction, *in vivo* release of ATP from urothelial cells was increased compared to controls (Akino et al., 2011). Contractions to ATP were reduced in obstructed urinary bladder of rats (Sjuve Scott et al., 2004). There is P2X3R up-regulation in interstitial cells of Cajal in an experimental rat model of partial bladder outflow obstruction (Li et al., 2013). In the pig, partial bladder outlet obstruction resulted in reduced contractions in response to electrical field stimulation as well as to purinergic agonists (Milicic et al., 2006), but in the rat partial outlet obstruction model, contractions to ATP were increased after 2 weeks and 3 months (Murakami et al., 2008). Patients with bladder outflow obstruction release higher amounts of ATP into the urine (Silva-Ramos et al., 2016).

#### Botulinum Toxin A (BTXA) and ATP Release

BTXA is increasingly being used to treat detrusor overactivity. In an early paper, it was shown that BTXA inhibited ATP as well as ACh release from parasympathetic nerves. Recently, BTX has been shown to inhibit ATP release from the urothelium (Chancellor et al., 2008; Ikeda Y. et al., 2012; Cruz, 2013). Daytime frequency, urgency, nocturia and pain were decreased by injection of BTXA into the human bladder. BTX is used to treat OAB syndrome and bladder hypersensory states (Apostolidis et al., 2005; Atiemo et al., 2005).

#### Chronic Alcohol Consumption and Bladder Function

Chronic consumption of ethanol impairs purinoceptor-mediated relaxation of detrusor smooth muscle of the rat (Calvert et al., 2002). Ethanol also alters neuronal P2XR.

#### Diabetic Bladder

Damage to human urinary bladder autonomic nerves and disturbances in micturition in diabetes have been recognised for many years. Urothelial release of ATP has been reported to play a role in bladder dysfunction in type 2 diabetes (Wang Z. et al., 2013). There is a transient increase in sensitivity of the STZ rat whole bladder preparation to α,β-meATP. The cholinergic component of nerve-mediated contractions was reduced in diabetic rat and rabbit bladders, but the purinergic component was increased (Mumtaz et al., 2006).

In STZ mice, the urinary bladder had weaker nerve-mediated contractions in response to electrical field stimulation in contrast to normal mouse bladder. Ca^2+^ regulation of ATP release may be impaired in diabetes. Increased adenosine- and ATP-mediated relaxant responses, together with increased ATP-mediated contractile responses were observed in bladders from 8-week STZ diabetic rats. The enhanced contractions of STZ-diabetic rat bladders to nerve stimulation and ATP peaked between 6 and 9 weeks, but dropped to control levels by 12–20 weeks (Daneshgari et al., 2006). There is also an increase in release of ATP in STZ female rat bladders (Munoz et al., 2008). There is upregulation of P2X1R in the bladder of STZ-induced diabetes in the early phase, but downregulation of the P2X2R (Liu et al., 2008). Bladder overactivity occurring 2 months after induction of diabetes with STZ was accompanied by enhanced expression of P2Y_2_ and P2Y_4_R (Suadicani et al., 2009).

#### Bacterial Infection

Bacterial infection of the bladder causes urgency, urinary incontinence and overactivity. Urine ATP concentrations were reduced during episodes of bacteriuria (Walsh et al., 2013). An ATP assay method has been employed for many years to test for bacteriuria in urine.

#### Bladder Cancer

The growth of bladder cancer cells is reduced by ATP, both *in vivo* and *in vitro* (Shabbir et al., 2008; Shabbir and Burnstock, 2009). Doxorubicin, used to treat bladder cancer, has side effects, such as increased urgency and urinary frequency. Quercetin, a plant-derived flavonoid, prevents bladder cancer in cells lines, by suppressing cell proliferation and inducing arrest of the cell cycle or cell death by inhibiting the activity of ecto-nucleotidases (Rockenbach et al., 2013). In humans with bladder papillary carcinoma urothelial differentiation was correlated with the expression and localisation of P2X3 and P2X5R (Sterle et al., 2014). High CD73 immunoreactivity was associated with reduced bladder tumour progression (Wettstein et al., 2015). P2X7R protein favoured survival of patients with bladder urothelial cell carcinoma (Hu J. et al., 2016).

#### Bladder Pain

In 1995, P2X3 homomultimer and P2X2/3 heteromultimer receptors were cloned and were localised on sensory nerve endings. Purinergic mechanosensory transduction was proposed (Burnstock, 1999), suggesting that release of ATP from urothelial cells as a result of distension stimulates P2X3 and P2X2/3R on suburothelial sensory nerve endings to activate high threshold nociceptive nerve fibres that reach the cortex pain centres (Burnstock, 2009). Distension of the bladder stimulates discharge in the nociceptive sensory nerves, which can be mimicked by ATP and can be inhibited by P2X3R antagonists (Vlaskovska et al., 2001). Stimulation of bladder P2X3R sensitises bladder afferent nerves, mimicking the sensitising effect of cyclophosphamide-induced cystitis. P2X3R are a potential target for therapeutic treatment of bladder pain. P2Y_2_R are also expressed on bladder sensory nerves and are claimed to mediate increased stimulation of P2XR-mediated activity, playing a role in bladder pain syndrome (IC) (Chen et al., 2010).

### Urethra

The principal non-adrenergic, non-cholinergic inhibitory transmitter to the urethra is NO, but a small component of purinergic neurotransmission is also involved (Andersson, 2001). ATP causes urethral relaxation, probably via P2Y_1_R, in pigs, guinea-pigs, rabbits and hamsters. The responses to non-adrenergic, non-cholinergic nerve stimulation were blocked by α,β-meATP, indicating that the P2X1R subtype was involved.

Sensory nerve fibres supplying the urethra may release ATP during axon reflex activity. Prostaglandins, produced following stimulation of P2YR, contribute to contractions of the urethra in pathophysiological conditions. In the rat, SC19220 (a prostaglandin E1 antagonist), lowered detrusor tone leading to increased bladder capacity and decreased voiding efficiency. Some sensory functions in the urethra are mediated by afferent fibres that express P2X3R (Canda et al., 2006). A review that discusses the therapeutic opportunities offered by K_ATP_ channels in the urethra is available (Kyle, 2014).

### Ureter

The ureter motor innervation is sparse, perhaps because peristaltic activity is not neurogenic, but myogenic. The dominant nerves in the ureter are sensory, confined mainly to the suburothelial plexus. These nerves contribute to vesicoureteral reflux that activates reflexes that modulate urine delivery to the bladder.

ATP constricts the pig ureter, while intravesical adenosine evoked relaxation via A_2B_R. In the ureter, ATP, α,β-meATP and adenosine evoked transient decreases in the frequency of peristalsis. In the rat ureter expression of P2X1R was shown on smooth muscle, while P2X5 and perhaps P2X7R are expressed on urothelium and P2X6R in the layer beneath the urothelium. P2X3R are localised on subepithelial sensory nerves that mediate nociception produced by release of ATP following distension of urothelial cells (Knight et al., 2002; Rong and Burnstock, 2004). Some ureter purinoceptors are likely to participate in long-term (trophic) events during development and regeneration, such as cell proliferation, differentiation, migration and cell death. The human ureter releases ATP in response to distension and human ureteric suburothelial sensory nerves express P2X3R (Calvert et al., 2008b). In a review of the pharmacology and physiology of the human ureter, it was proposed that purinergic receptors may prove to be analgesic targets to treat ureteral colicky pain and to facilitate ureteral stone passage (Canda et al., 2007).

Seven days after unilateral ureteral obstruction of wild-type mice, it was shown that there was increased expression of P2X7R associated with fibrogenic responses and inflammation in the cortex. However, in P2X7R KO mice, the alterations seen in the wild-type mice were not present. It was suggested that P2X7R antagonists may play a role in preventing renal interstitial fibrosis. In A_2A_R KO mice with unilateral ureteral obstruction, there was a substantially increased progression of renal interstitial fibrosis (Xiao et al., 2013).

## Diseases of the Liver

In the liver, the two epithelial cell types that secrete bile, i.e., hepatocytes and cholangiocytes, express purinoceptor in the plasma membrane. Modulation of the release of ATP and purinergic signalling may be novel strategies to manage cholestasis and other bile flow disorders. Both quiescent and activated hepatic stellate cells (HSC) express purinergic receptors: P2Y_2_ and P2Y_4_R on quiescent and P2Y_6_R on activated HSC. P2YR on satellite cells could be a therapeutic target to treat or prevent liver fibrosis. Reviews concerned with purinergic signalling in liver disease are available (Burnstock et al., 2014b; Vaughn et al., 2014).

### Liver Injury, Inflammation, Immune Regulation and Repair

Purinergic signalling regulates the immune response in the liver. In A_1_R KO mice α-naphthylisothiocyanate-induced cholestatic liver injury was decreased. CD39-deficient mice treated with adenosine, probably via A_2A_R, were protected from reperfusion injury (Sun et al., 2011). A_1_R antagonists abolished ischaemic preconditioning. After the resection of bile-duct-ligated cirrhotic livers, an A_2A_R agonist improved liver function (Iskandarov et al., 2016). Inhalation of high concentrations of hydrogen protects against ischaemia/reperfusion injury through A_2A_R activation (Li H. et al., 2017). Adenosine, via A_2B_ or A_3_R, accelerates the cell cycle during partial hepatectomy-induced liver regeneration in rats (Mendieta-Condado et al., 2007). In mice, ATL-146e, a selective A_2A_R agonist, prevented concanavalin A-induced acute liver injury.

P2Y_2_R activation in mice makes a major contribution to endotoxin-induced acute liver injury (Samuel et al., 2010). In mice with acute liver injury, P2Y_2_R mediate neutrophil infiltration, regulating immune responses associated with death of hepatocytes. P2Y_2_R antagonists might be useful to treat inflammatory liver disease (Ayata et al., 2012). A review discusses the role of purinergic signalling in sterile liver injury (Oliveira et al., 2013).

Infusion of ATP-MgCl_2_ was shown early to improve hepatic function and survival following hepatic ischaemia and after reperfusion. During ischaemia there is a substantial loss of ATP from hepatocytes. Resistance of hepatocytes to hypoxia is promoted by P2Y_2_R. In mice, CD39 deletion from natural killer (NK) cells reduced hepatic ischaemia/reperfusion injury, indicating that during liver regeneration ATP modulates NK cell function. UTP acting via P2Y_2_ and/or P2Y_4_R before induction of ischaemia attenuates post-ischaemic hepatocyte apoptosis resulting in a reduction of liver damage (Ben-Ari et al., 2009). Platelet aggregation triggered by ADP may have a role in ischaemia reperfusion injury (Schulte am Esch et al., 2010).

ATP released from sympathetic nerves and from hepatocytes, may participate in the regulation of liver repair. In P2Y_2_R KO mice, hepatocellular proliferation is impaired, indicating that ATP has a trophic role in liver regeneration and growth after injury (Thevananther et al., 2008). ATP release following partial hepatectomy in rats regulates liver regeneration. Apyrase (CD39/NTPDase1) reduces extracellular ATP allowing NK cells to play a role in the regulation of the immune response and to improve liver regeneration (Graubardt et al., 2013). After partial hepatectomy, liver regeneration is enhanced by the K_ATP_ opener, diazoxide (Nakagawa et al., 2012).

Hepatocyte lipoapoptosis contributes to hepatic inflammation in lipotoxic liver injury. Pannexin1 may contribute to hepatic inflammation by increasing ATP release in lipotoxic liver injury (Xiao et al., 2012). Acetaminophen (APAP), used to reduce pain and fever, may damage hepatocytes. In mouse APAP-induced inflammation models, full injury following an overdose involved P2X7R activation (Amaral et al., 2013). P2X7R activate hepatic caspase-1 as well as the migration of neutrophils into the liver, suggesting that ATP may play a major role in the development of inflammasomes following overdose of APAP (Hoque et al., 2012). A438079, a P2X7R antagonist, is protective against APAP-induced liver injury (Xie et al., 2013). P2X4R are involved in liver regeneration after partial hepatectomy in mice (Besnard et al., 2016). P2X1R-regulated IL-22 secretion is required for liver regeneration (Kudira et al., 2016).

### Fibrosis and Hepatic Stellate Cells (HSC)

Liver fibrosis followed by cirrhosis is a common cause of liver failure. HSC are the main fibrogenic cells of the liver, which express nucleotide receptors that are functional (Kruglov et al., 2007) and mediate phospholipase D activity (Benitez-Rajal et al., 2006).

Adenosine A_2B_R play pro-fibrotic roles in human HSC (Zhong et al., 2007). During fibrosis, HSC proliferate and undergo senescence and A_2A_R mediate both these key processes, suggesting that A_2A_R antagonists are potential antifibrotics (Ahsan, 2011). A_2A_R contribute to the pathogenesis of hepatic fibrosis and A_2A_R antagonists may reduce ethanol-induced stellate cell activation and fibrosis (Szuster-Ciesielska et al., 2012; Chiang et al., 2013). MRS1754, an A_2B_R antagonist, reduced hepatic collagen deposition during fibrosis progression (Stoll et al., 2012).

Activation of P2Y_2_ and P2Y_6_R regulates procollagen-1 transcription and may be targets to treat or prevent liver fibrosis, thereby avoiding cirrhosis and chronic liver failure. PPADS, a P2R antagonist, inhibited HSC proliferation and prevented the development of non-biliary liver fibrosis (Dranoff et al., 2007). NTPDase2 is a preferential ATPase that greatly influences inflammation and biliary type fibrogenesis. Ecto-5′-nucleotidase (CD73) gene expression in HSC and portal fibroblasts increased during myofibroblastic differentiation and is a therapeutic target for antifibrotic therapy (Fausther et al., 2012).

Human platelet ATP release contributes to reduced type I collagen production and HSC activation *in vitro* (Ikeda N. et al., 2012). In progressive fibrosis, eicosapentaenoic acid replenishes hepatic levels of ATP leading to a reduction of steatosis and inflammation (Jia et al., 2012). Blockade of the P2X7R-NLRP3 inflammasome axis is considered to be a potential therapeutic target for liver fibrosis (Jiang et al., 2017).

### Cirrhosis

Liver injury induced by alcohol is associated with enhanced inflammatory responses and adenosine, acting via A_2A_R, may prove to be an effective strategy for reducing liver injury (Pritchard et al., 2011). During fulminant hepatitis, A_2A_R contribute to the anti-inflammatory actions that limit liver damage (Choukèr et al., 2008). In cirrhosis, platelet dysfunction is partly mediated by purinergic signalling. Cerebral A_1_R are involved in liver cirrhosis (Boy et al., 2008). Ectonucleotidase NTPDase 2 is down-regulated in biliary cirrhosis. It has also been claimed that endogenous A_1_R activation may protect mice against liver injury induced by acute ethanol by reducing oxidative stress and decreasing the accumulation of lipid (Yang et al., 2013). ATP-MgCl_2_ was used early to improve survival following massive hepatectomy in cirrhotic rats and adenosine partially reversed cirrhosis induced by carbon tetrachloride. In alcoholic liver disease, ATP and uric acid mediate inflammatory cross-talk between immune cells and hepatocytes (Petrasek et al., 2015). A_2A_R are involved in the pathogenesis of hepatic cirrhosis (Chan et al., 2006).

### Hepatitis

*In vitro* infection by duck hepatitis B virus, *Rous sarcoma* virus, and hepatitis delta virus was inhibited by suramin. Sympathetic nerves influence immune-mediated experimental hepatitis (Neuhuber and Tiegs, 2004) and ATP released as a cotransmitter with NA is probably involved. In autoimmune hepatitis P2X7R regulate NKT cells (Kawamura et al., 2006). P2X7R activation participates in hepatitis delta and hepatitis B virus infection of human hepatocytes (Taylor and Han, 2010). Leptin-induced GLUT4 function in stellate cells in non-alcoholic steatohepatitis is mediated by P2X7R (Chandrashekaran et al., 2016). Chronic hepatitis C virus infection evokes progressive liver disease, exhibiting cirrhosis, insulin resistance, fibrosis and finally liver cancer. P2X4 and P2X7R may be a major component of the purinergic signalling complex in hepatitis C virus-induced liver pathogenesis (Manzoor et al., 2016). In rats, A_2A_R stimulation inhibited hepatocyte lipotoxicity and non-alcoholic steatohepatitis (Imarisio et al., 2012). Adenosine, via A_2A_R, controls NKT-cell-dependent hepatitis induction (Subramanian et al., 2014). Purinergic mechanisms involved in autoimmune hepatitis have been reviewed (Kapila et al., 2013).

### Liver Transplantation

Human liver can be maintained successfully under hypothermic conditions for a maximum of 10 h using adenosine at high concentrations, although overcoming ischaemic damage is a major obstacle. However, infusion of ATP can preserve cells injured sublethally by enhancing their recovery following ischaemic injury and purinergic receptor antagonists prevent cold preservation-induced cell death. Upregulation of CD39 post-adenoviral infection prolongs transplant graft survival. After transplantation regeneration of the donor liver is important and ATP, via P2Y_2_R, activates hepatocyte cell cycle progression and proliferation *in vitro* and modulates growth factor activities *in vivo* (Thevananther et al., 2004). A_2A_R stimulation down regulated adhesion molecules, proinflammatory cytokines and ultimately improved liver function following liver transplantation in rats (Tang et al., 2010). In post-transplant allografts, purinergic signalling is used to predict as well as monitor progression of fibrosis and rejection. Blood from acute rejection patients showed raised intracellular levels of ATP in CD4^+^ lymphocytes (Qu et al., 2017).

### Liver Cancer

Primary liver malignant tumours are subdivided in to hepatocarcinoma, bile duct carcinoma (cholangiocarcinoma) and hepatocholangiocarcinoma. ATP increases calcium uptake by rat hepatoma cells, probably via P2Y_2_ or P2Y_4_R subtypes. There is upregulation of P2Y_2_R in human hepatocellular carcinoma cells (Tak et al., 2016), which mediates proliferation and migration of the cells (Xie et al., 2014). Carcinoma-specific expression of P2Y_11_R make a major contribution to ATP-induced signalling that controls cell migration in human hepatocellular carcinoma cells (Khalid et al., 2017). CD39 KO mice showed an increased incidence of spontaneous and induced hepatocellular carcinoma. Intra-arterial injection of an inhibitor of ATP production was suggested as a novel liver cancer therapy. The effects of ATP infusions *in vivo* on rat hepatocarcinomas have been investigated (Frontini et al., 2011). P2X3R over-expression is involved in poor recurrence-free survival in hepatocellular carcinoma patients and identifies the P2X3R as a potential therapeutic target (Maynard et al., 2015). Inhibition by adenosine of hepatoma cell growth was reported early.

The A_3_R agonist, CF101, inhibited liver metastasis (following colon carcinoma) (Bar-Yehuda et al., 2008). CF102, another selective A_3_R agonist, has anti-inflammatory and anti-tumour effects on the liver and was studied in a clinical trial for hepatocellular carcinoma patients (Stemmer et al., 2010). A_2B_R are strongly expressed in human hepatoma cellular carcinoma (Xiang et al., 2006). Mouse regulatory T cell CD39 expression mediated inhibition of NK cell activity and promoted hepatic metastatic tumour growth (Sun et al., 2013). Liver metastasis from colorectal cancer is one of the main causes of cancer-related morbidity and ATP-chemotherapy may effectively treat initially unresectable colorectal liver metastasis (Hur et al., 2012). In hepatocellular carcinoma, up-regulation of ATP-binding cassette transporter genes is mediated by cellular microRNAs (Borel et al., 2012).

## Diseases of the Reproductive System

Reviews are available about purinergic signalling in the reproductive system in both health and disease (Burnstock, 2014c; Gorodeski, 2015).

### Disorders of the Male Reproductive Tract

#### Erectile Dysfunction

Abnormalities in purinergic signalling, including impaired ATP-mediated cavernosal relaxation, may contribute to erectile dysfunction associated with prostate enlargement and diabetes and may provide a target for therapy (Hupertan et al., 2012; Wen and Xia, 2012). Normal penile erectile function involves a fine balance between contraction and relaxation in the corpus cavernosum smooth muscle. The strong relaxation induced by ATP via P2YR on human corpus cavernosum is comparable to that produced by NO, thus ATP together with an NO donor may prove to be effective for erectile disorders (Hupertan et al., 2012). ATP released from sympathetic nerves leads to relaxation of cavernosum smooth muscle via P2Y_4_R, whereas ADP, after breakdown of ATP released from endothelial cells, acts via P2Y_1_R on endothelial cells to produce relaxation via NO (Calvert et al., 2008a). P2XR might also be involved (Gur et al., 2007; Phillips et al., 2014).

In anaesthetised dogs, ATP and adenosine induce penile tumescence, probably via A_2_R; pelvic nerve stimulation also produced tumescence. Impaired adenosine signalling via A_1_R contributes to erectile dysfunction (Ning et al., 2012a). Elevated adenosine signalling, via A_2B_R, may contribute to priapism, where there is persistent penile erection lasting at least 4 h without sexual excitation (Dai et al., 2009; Ning et al., 2012b). A review highlights adenosine signalling in penile tissue as a potential therapeutic target to treat erectile disorders (Wen and Xia, 2012). Impaired erectile function occurs in CD73 KO mice resulting in decreased endogenous adenosine. Corpus cavernosum from men suffering from vasculogenic erectile dysfunction show lower ectonucleotidase CD39 activity resulting in ATP accumulation. Human corpus cavernosum relaxation by P2R agonists was substantially reduced in erectile dysfunction patients (Faria et al., 2010). ATP release from cavernosal tissue increased in patients following prostatectomy. Activating ATP-mediated pathways may restore erectile function in diabetics where there is impaired NO bioavailability. It has been suggested that P2X3 antagonists may improve recovery of erectile function (Li C.L. et al., 2015).

#### Male Fertility and Contraception

ATP increases the fertilising potential of sperm in humans and is used to treat spermatozoa during *in vitro* fertilisation. In P2X1R KO mice fertility was diminished with decreased number of spermatozoa in the ejaculate (Mulryan et al., 2000). This raises the possibility that P2X1R antagonists would provide a safe and effective contraceptive (White et al., 2013). In rat testes, during spermatogenesis there is differential, stage-dependent immunostaining for P2XR subtypes (Glass et al., 2001), suggesting purinergic targets for both fertility and contraception. ATP triggers the acrosome reaction via P2X7R in human spermatozoa (Torres-Fuentes et al., 2015). Purinergic signalling plays a role in the maturation of sperm cells in the testes. When the selective P2X1 and P2X3R desensitiser, α,β-meATP, was injected into the cauda epididymis, fertility in male rats was impaired. Adenosine stimulates human sperm motility via A_2_R.

#### Prostatic Hyperplasia

Purinergic compounds have been suggested for the therapeutic treatment of benign prostatic hyperplasia (Andersson et al., 2002). Injection of BTX into the prostate, which reduces ATP and ACh release, treats bladder obstruction hyperactivity, by decreasing prostate size and as such improving urine flow rate (Chuang and Chancellor, 2006).

### Disorders of the Female Reproductive Tract

Regulating the proliferation of ovarian granulosa cells as well as steroidogenesis contributes to ovarian pathophysiology, since in rats with polycystic ovarian syndrome, theca hyperplasia occurs (Salvetti et al., 2009). The hydrolysis of ADP and ATP was reduced by ovariectomy and oestradiol replacement therapy (Pochmann et al., 2004). Ovarian tumours arise largely from the surface of squamous-to-cuboid mesothelium covering the ovary. ATP stimulated mitogen-activated kinase in neoplastic and pre-neoplastic surface epithelium, suggesting that co-released ATP from sympathetic nerves may contribute to the regulation of cell proliferation in neoplastic epithelial cells from the surface of the ovary (Choi et al., 2003).

In the human fallopian tube, ATP-mediated contractions are increased during acute purulent inflammation, probably as a result of upregulation of P2X1 and P2X2R (Ziganshin et al., 2008). It has been claimed that targeting P2X7R may lead to new treatments to prevent uterine contractions in preterm deliveries. A naturally occurring P2X7 splice variant, the P2X7jR, blocks P2X7R-mediated actions (Feng et al., 2006). It is co-expressed with P2X7R in female reproductive tract epithelia. The P2X7j isoform hetero-oligomerises with the P2X7R and co-expression of P2X7R and P2X7jR blocks ATP-induced pore formation, and abolishes agonist-induced apoptosis. P2Y_2_R agonists may be a non-hormonal alternative therapy for treating vaginal dryness in post-menopausal women. ATP is considered as a therapeutic target to control uterine activity during difficult labours (Zafrah and Alotaibi, 2017).

The P2X7R contributes to the control of cervical infections. P2X7R-mediated activation of cervical epithelial cells inhibits *Chlamydia* and mycobacteria infection (Darville et al., 2007). ATP regresses endometriosis in a rat model (Zhang C. et al., 2016). Adenosine in the placenta mediates the placental disturbances induced by alcohol, perhaps contributing to the pathogenesis of foetal alcohol syndrome (Acevedo et al., 1997). Adenosine protects vaginal epithelial cells from *T. vaginalis* cytotoxicity (Menezes and Tasca, 2016). Plasma adenosine is raised in hyperemesis gravidarum (severe morning sickness) and serves as a prejunctional modulator of sympathetic neurotransmission, which limits further progression of this pregnancy-related disease (Yoneyama et al., 2004).

#### Preeclampsia

ATP infusion in pregnant rats involved an inflammatory response that occurs in preeclampsia (Spaans et al., 2014a). Elevated placental adenosine signalling contributes to the pathogenesis of preeclampsia (Iriyama et al., 2015). Hypoxia stimulates ATP release, which is rapidly broken down to adenosine by ectonucleotidases and women with preeclampsia and their foetuses have increased circulating adenosine concentrations. There is elevation of adenosine A_2A_R expression in placental biopsies, villous explants and placental microvillous membranes (von Versen-Höynck et al., 2009). In preeclampsia A_2B_R on microvascular endothelial cells have also been implicated (Escudero et al., 2008). Reduced adenosine-mediated angiogenesis in preeclamptic pregnancies may be associated with hypertension development in the offspring (Escudero et al., 2014). The interaction between A_2A_R and the angiotensin system may be involved in the early growth of the placenta. A_2A_R expression is raised in pre-eclampsia, perhaps as a consequence of poor placental perfusion in preeclampsia (Kurlak et al., 2015). Elevation of ADA activity in women with preeclampsia may contribute to their increased levels of uric acid and pro-inflammatory immune activity (Giorgi et al., 2016). Release of ATP increases in preeclampsia following hypoxia and oxidative/nitrative stress, which acts on P2X4R to influence homeostasis of the placenta (Roberts et al., 2007). There is deficient spiral artery remodelling and trophoblast invasion in preeclampsia, both of which may be inhibited by ATP-induced activated macrophages (Spaans et al., 2014b). It was concluded from a study of the relationship between the foeto-placental adenosine release and utero-placental circulatory insufficiency in pregnancies featuring preeclampsia, that foetal plasma adenosine increases before utero-placental insufficiency induces generalised foetal hypoxia (Yoneyama et al., 1996).

### Malignant Cancer of Reproductive Organs

#### Prostate Cancer

Prostate cancer is the second most common male cancer and the third leading cause of cancer death. Prostate cancer cells are sensitive to extracellular ATP. ATP and adenosine inhibit the growth of human prostate cancer cells (Lertsuwan et al., 2017), identified at that time to act via P2Y_1_, P2Y_2_ and/or P2Y_4_, P2Y_6_ and P2Y_11_R subtypes. Activation of P2Y_1_R inhibited growth and induced cell death of prostate cancer PC-3 cells and P2Y_1_R agonists were claimed to be therapeutically promising for prostate cancer (Wei et al., 2011).

However, prostate tumour cells were shown later to also express P2X4, P2X5, and P2X7R in PC-3 cells and P2X4 and P2X5R in DU145 cells. ATP inhibited tumour cell growth, but not by UTP or adenosine, while 2′(3′)-*O*-(4-benzoylbenzoyl) ATP increased apoptotic cell death in PC-3 cells, probably via P2X7R. CD73 KO mice resist prostate carcinogenesis and CD73 promoted *de novo* prostate tumorigenesis. Anti-CD73 monoclonal antibodies decreased tumour growth and metastasis in the prostate (Stagg et al., 2012).

There are many polymorphisms of the P2X7R (Fuller et al., 2009), which, as well as resulting in loss of function, alter receptor activity. Cytolytic P2X7R expression was found in 116 prostate cancer pathology specimens (Slater et al., 2004). In normal tissues from patients with no evidence of cancer, P2X7R were not expressed, suggesting that the appearance of P2X7R is an early marker of prostate cancer.

Prostate tumour cell proliferation is inhibited by adenosine. A_3_R activation by IB-MECA inhibited proliferation of prostate cancer cells and induced cell cycle arrest and apoptosis (Aghaei et al., 2012). Activation of A_3_R suppressed prostate cancer metastasis (Jajoo et al., 2009). The ATP synthase β subunit also plays a role in prostate cancer metastasis (Li W. et al., 2017).

#### Breast Cancer

Inhibition of growth of human breast cancer cells by ATP was shown for the first time in 1993. Chemotherapeutic ATP release from breast tumour cells of mice increased tumour regression through apoptosis and it was suggested that P2Y_2_ and/or P2Y_4_R were involved. Oestrogen, acting via oestrogen receptor α, promoted proliferation of breast cancer cells by down-regulating expression of P2Y_2_R and reducing P2Y_2_R-induced increase in [Ca^2+^]_i_ (Li et al., 2011). P2Y_2_R activation by ATP released from cancer cells induces the invasion of metastatic breast cancer cells (Eun et al., 2015). Up-regulation of P2Y_6_R occurs in the mesenchymal phenotype of breast cancer cells and inhibition of P2Y_6_R may be a useful therapeutic target for metastasis of breast cancer (Azimi et al., 2016; Ma X. et al., 2016).

Antibody therapy with anti-CD73 inhibited breast tumour growth and metastasis (Stagg et al., 2010). Bisphosphonates are also effective inhibitors of breast cancer (Fehm et al., 2012). Proteomic analysis of human breast carcinoma revealed upregulation of ATP synthase in tumours and the ATP synthase inhibitor, aurovertin B, inhibited proliferation of several breast cancer cell lines (Huang et al., 2008). Malignant breast carcinoma cells release ATP making the pre-metastatic environment suitable for micro-metastasis in lymph nodes and associated afferent lymph vessels (Kawai et al., 2008).

In breast tumour cells ATP increased [Ca^2+^]_i_ and high concentrations produced apoptosis via P2X7R. Activation of P2X7R in the human breast cancer cell line, T47D, increased cell migration and development of metastases, suggesting that P2X7R antagonists may have therapeutic roles (Xia et al., 2015). The role of hypoxia in the regulation of tumour progression has been debated. However, P2X7R expression is increased by hypoxia and hypoxia-driven increase in P2X7R enhances tumour cell invasion and migration. Silencing of P2X5R inhibited cell proliferation and may be a new mechanism to target cancer metastasis.

A_1_ and A_3_R mRNA are expressed on human breast tumours (Panjehpour et al., 2012). Adenosine induces tumour cell proliferation and migration of T-47D and MCF-7 breast carcinoma cell lines (Mujoomdar et al., 2004). MDA-MB-231, a human breast cancer cell line, expressed A_2B_R, which mediated cell proliferation and A_2B_R inhibition slowed growth of breast tumours (Cekic et al., 2012). A_3_R agonists reduced bone metastasis of breast cancer, suggesting a therapeutic approach to bone-residing breast cancer (Varani et al., 2013).

#### Cervical Cancer

HeLa cells from human cervical cancer are used to study purinergic signalling involvement in cancer. Activation of P2Y_2_ R with UTP and ATP caused proliferation of HeLa cells. P2Y_4_ and P2Y_6_ R expression increased during proliferation. Stimulation of P2Y_1_R on HeLa cells triggered epidermal growth factor receptor mitogen signalling and P2Y_1_ antagonists reduced proliferation. Permeabilisation of cervical cancer cells to a cytotoxin is activated by P2YR (Bukhari et al., 2015). P2Y_6_R activation induces HeLa cell migration (Gendaszewska-Darmach and Szustak, 2016). Oestrogen reversed the apoptotic activity mediated by P2X7R in normal human cervix, but not in cervical epithelial cancer cells. A truncated P2X7R variant (P2X7-j), expressed in cervical cancer cells, antagonised the P2X7R through hetero-oligomerisation. Gentle mechanical stimulation released ATP from HeLa cells. The presence of ectonucleotidase in human cervical cancer cell regulates the levels of nucleotides, limiting their effects (Beckenkamp et al., 2014).

### Ovarian Cancer

ATP raised [Ca^2+^]_i_ and stimulated growth of SKOV-3 and OVCAR-3 human ovarian carcinoma cells. ATP may act as a messenger to control the ovarian epithelial cell cycle through P2Y_2_R on human ovarian cancer cells. A_2_R antagonists inhibited angiogenic activity of human ovarian cancer cells. The treatment of human ovarian carcinoma with cisplatin in the presence of ATP results in additive cytotoxicity (Rotte et al., 2010). P2X7R were highly expressed in both human ovarian tumours and ovarian cancer cell lines and paracrine release of ATP acts on P2X7R to cause proliferation of ovarian cancer cells (Vazquez-Cuevas et al., 2014).

#### Uterine Cancer

P2Y_2_R play a role in the control of the cell cycle and in the suppression of proliferation of human endometrial carcinoma cells. The P2X7R has been used as a biomarker for uterine epithelial cancers. There is decreased P2X7R expression on endometrial epithelium in pre-cancerous and cancer cells (Li et al., 2007, 2009). Activation of P2X7R-dependent apoptosis has a chemotherapeutic growth-preventive effect on pre-cancerous and early cancerous epithelial lesions (Fu et al., 2009; Gorodeski, 2009). Loss of CD73 in endometrial cancer allows for tumour progression (Bowser et al., 2016). A higher proportion of ADA2^∗^1/^∗^1 genotype was observed in women with uterine leiomyomas (Gloria-Bottini et al., 2016).

## Skin Diseases

Reviews about purinergic signalling in the skin during health and disease have been published (Burnstock et al., 2012b; Geraghty et al., 2016). Changes in P2R subtype expression occurs in the epidermis during proliferative disorders, such as psoriasis and scleroderma, and P2Y_2_R may be a novel target to treat these disorders. ATP plays an important role in wound healing, the defence response, innate immunity and inflammation in the skin and might be an important therapeutically in psoriasis and scleroderma.

### Psoriasis

Psoriasis is a chronic skin disease involving epidermal hyperproliferation. ATP and parathyroid hormone-related protein increased proliferation in HaCaT cells and may account for the hyperproliferation that occurs in psoriasis. P2Y_2_R contribute to epidermal homeostasis and indicate a possible therapy for psoriasis. P2Y_11_R mediate IL-6 production in human keratinocytes, which is important in psoriasis (Ishimaru et al., 2013). P2X7R signalling induces inflammation leading to differentiation of Th17 lymphocytes, which are involved in the pathogenesis and potential treatment of psoriasis (Killeen et al., 2013). P2X7R play a role in shaping the inflammatory microenvironment in psoriasis (Lioi et al., 2015). Strong P2X7R expression is confined to the basal layer cell membrane, while P2Y_1_R were expressed all through the psoriatic epidermis.

In the blood of psoriasis patients, there are high levels of adenosine. Adenosine raised cyclic AMP levels in lesions of epidermis from psoriasis patients. There is a defective purine nucleotide synthetic pathway in patients with psoriasis and nucleotide metabolism is altered in psoriatic keratinocytes. In psoriasis patients, ADA levels were significantly elevated in both serum and epidermis. It was concluded from a clinical trial that caffeine, a P1R antagonist, is an inexpensive, safe and effective treatment for psoriasis. The role of adenosine as an endogenous mediator of the pathogenesis of psoriasis has been reviewed (Festugato, 2015) and clinical trials for the use of A_3_R agonists for the treatment of psoriasis described (Borea et al., 2015; Kofoed et al., 2015). A_2A_R are upregulated in psoriasis and A_2A_R agonists may counteract inflammation in this disease (Merighi et al., 2017). A_2B_R are also expressed by human epidermal keratinocytes and their expression is reduced in psoriasis (Andrés et al., 2017).

### Scleroderma

Scleroderma encompasses a spectrum of disorders that cause dermal fibrosis and systemic sclerosis (SSc). SSc is a severe disease of the connective tissue of affected organs, including the skin. Adenosine A_2A_R contribute to the pathogenesis of dermal fibrosis and may be a therapeutic target to treat and prevent dermal fibrosis in scleroderma (Chan and Cronstein, 2010). Fibroblasts from SSc patients had a high rate of spontaneous IL-6 release, which was increased by ATP stimulation. SSc fibroblasts expressed mRNA for P2X3, P2X4, P2X7, P2Y_1_, P2Y_2_, P2Y_4_, and P2Y_6_, suggesting a possible therapeutic role for P2R antagonists in SSc patients via modulation of fibroblast function.

### Skin Inflammation

The skin is an immune defence organ. Chemical, immune-specific or physical insults evoke increased expression of proinflammatory mediators, in particular keratinocytes release chemokines. Release of ATP is associated with inflammation of the skin, as well as increase in expression of, in particular, P2X7 and perhaps P2Y_1_R, and subsequent release of proinflammatory cytokines. Skin inflammation is reduced by P2X7R antagonists.

In ectonucleotidase (CD39) KO mice, rapid ATP release from keratinocytes is triggered by irritant chemicals causing exacerbated skin inflammation. Ultraviolet (UV) radiation evokes ATP release from keratinocytes and P2Y_6_R mediate UV radiation-induced inflammatory responses (Takai et al., 2011). ATP, when released after trauma or infection, may enhance immunoresponses and P2R agonists may increase vaccine efficacy. A_1_ and A_2_R may function as cutaneous neurogenic pro-inflammatory mediators. Human skin keratinocytes infected by *Staphylococcus aureus*, which releases α-toxin, exhibited a transient reduction in cellular ATP levels (Suriyaphol et al., 2009). P2X3 and P2X2/3 nociceptive receptors on sensory nerve endings are increased in inflamed skin and antagonists to these receptors have been developed as analgesics.

### Wound Healing

Topically applied A_2B_ and particularly A_2A_R agonists promote cutaneous wound healing in both healthy and diabetic conditions. A_2A_R agonists also promote collagen production by dermal fibroblasts. Adenosine inhibits proliferation of vascular smooth muscle cells via A_2_R activation. A review discusses the involvement of A_2A_ and A_2B_R signalling in wound healing and fibrosis (Shaikh and Cronstein, 2016).

ATP and ADP also play a role in wound healing. ATP, released by damaged cells and physiologically during gentle mechanical stimulation, contributes to wound healing, tissue repair and regeneration. Cutaneous wound healing is accelerated by Mg-ATP, probably by increasing synthesis of vascular endothelial growth factor. Release of ATP from platelets and other cells during wound healing results in an increase in [Ca^2+^]_i_ in keratinocytes, associated with epidermal growth and differentiation. ATP improves ischaemic skin flap survival after surgery, while P2R antagonists accelerate barrier repair.

There is acute inflammation in the initial phase of wound healing. ATP is involved in the development of inflammation through P2X7R-mediated production and release of cytokines from immune cells. The presence of P2X7R on immune cells mediates killing of intracellular pathogens by stimulating apoptosis of the host macrophage, chemo-attraction and cell adhesion. In contrast, adenosine has anti-inflammatory effects. ATP released following infection and trauma acts as an endogenous adjuvant to increase the immune response and P2R agonists may enhance the efficacy of vaccines.

Wound healing also involves new vessel growth (angiogenesis) and both ATP and adenosine contribute to cell proliferation and migration during angiogenesis. In cultured porcine artery smooth muscle cells, ADP and ATP stimulate DNA synthesis and cell proliferation. ATP and UTP are mitogenic in human vascular smooth muscle cells and P2Y_2_ and P2Y_4_R might be involved.

Wound healing is delayed in denervated wounds. In a rat model of this, P2X5, P2X7, P2Y_1_, and P2Y_2_R expression was altered in the epidermis. P2Y_1_R expression was increased in the basal proliferating layer of keratinocytes in the regenerating epidermis, while P2Y_2_R were significantly decreased. ATP and UTP, probably via P2Y_2_R, increased proliferation of the MCS-P5 murine keratinocyte cell line, and enhanced wound healing in mice (Kehasse et al., 2013; Jin et al., 2014). ATP release and P2YR signalling mediate electric field-stimulated directional keratinocyte migration (Riding and Pullar, 2016). A review discussed the clinical applications of purinergic compounds to enhance wound healing (Gendaszewska-Darmach and Kucharska, 2011).

### Warts

Warts are caused by human papillomavirus infection of epidermis basal keratinocytes. P2X5R staining was increased in warts. P2X7R immunoreactivity was found in hyperkeratotic areas of the stratum corneum and in nuclei of koilocytes in the wart suprabasal layers. The nuclei positive for P2X7R were shrunken, showing much more intense P2X7R staining. The expression of P2X7R in the nucleus of human papillomavirus-infected cells was associated with disruption of the cellular machinery. P2X7R agonists may be used to trigger apoptosis in these virally infected cells. P2X5 and P2X7R are being explored for the treatment of warts.

### Allergy

Contact allergen sensitisation involves immune system activation by endogenous danger signals. P2X7R KO mice exhibit resistance to contact hypersensitivity. P2X7R KO dendritic cells do not induce sensitisation in response to contact allergens or release IL-1β in response to ATP. This suggests that P2X7R are crucial for release of ATP from skin in response to contact allergens. Interference with P2X7R signalling could be a therapeutic approach to prevent allergic contact dermatitis.

### Hailey–Hailey and Darier Diseases

These are autosomal dominant skin disorders and are characterised by epidermal keratinocyte dissociation (acantholysis) at the epidermis suprabasal layer. In lesions P2Y_2_R are not localised on acantholytic cells, but P2X7R appear in the plasma membranes, potentially mediating apoptosis.

### Barrier Function

The skin protects the water-rich internal organs from environmental dryness, with the stratum corneum being critical to the water-impermeable barrier. Topical application of ATP and α,β-meATP via P2XR delayed barrier recovery, damaged by surfactant, organic solvent or tape stripping. P2R antagonists, however, accelerated barrier repair. In mice skin wounds healed faster following treatment with ATP-encapsulated fusogenic lipid vesicles rather than with lipid vesicles. PPADS, a P2XR antagonist, accelerates skin barrier repair and prevented epidermal hyperplasia.

### Burns

There is increased concentration of adenosine in burn blister fluid, and depletion of ATP in skin after thermal injury. ATP has a protective effect against skin burns. ATP-MgCl_2_ administration following burn injury reversed the damage to the intravascular clearing of lipid emulsions within the reticuloendothelial immune system. Glucose metabolism was affected by ATP in thermally injured skin.

Burn injury produces severe pain and the relieving effects of tetramethylpyrazine, a Chinese medicine, are claimed to be due to it acting as a P2X3R antagonist (Gao Y. et al., 2010). Skin P2X3R expression was increased on sensory nerve terminals in first and second degree burns, but, following treatment with tetramethylpyrazine, P2X3R expression was reduced. P2X3R may play a role in the short-lasting thermal hyperalgesia induced by mild heat injury (Füredi et al., 2010). After a superficial skin burn, adenosine reduced the skin area showing hypersensitivity.

### Pain

Skin pain is associated with changes in sympathetic nerve activity and ATP released from rat sympathetic nerves as a cotransmitter with NA may modulate cutaneous nociceptors. P2X3R are expressed on sensory neurons in DRG, nodose and trigeminal ganglia that innervate cutaneous tissues. ATP and ADP stimulate skin afferent nerve terminals and skin cell damage stimulates nociceptive sensory nerves via ATP release. In rat skin with carrageenan-induced inflammation, nociceptors can be selectively activated with α,β-meATP, indicating the involvement of P2X3R. ATP contributes to the enhanced sensitivity of inflamed skin nociceptors, leading to heat hyperalgesia. P2X3R antagonists are anti-nociceptive. UV light evokes hypersensitivity of skin to ATP-induced pain. After subcutaneous bee venom injection, there is reduction of prolonged pain by PPADS, and it was suggested that stimulation of P2XR in the spinal cord contributes to cutaneous pain. The terminals of nociceptive neurons in the skin are targets for P2X3R antagonists. UTP sensitises a subpopulation of cutaneous C-fibre nociceptors to mechanical stimuli. There is increased P2X3R expression on calcitonin gene-related peptide-positive sensory fibres during the growth of tumours, mediating nociception. Increased expression of skin glial cell neurotrophic factor increases the mechanical sensitivity of nociceptive afferents expressing P2X3R.

Ecto-5′-nucleotidase (CD73) is located on nociceptive terminals and epidermal keratinocytes in the epidermis, which, by hydrolysing AMP to adenosine, is suggested to be anti-nociceptive. Blockade of peripheral P2Y_1_R prevents thermal hyperalgesia (Kwon et al., 2014). Mechanical allodynia is induced by α,β-meATP, which sensitises P2X3R on cutaneous nociceptive sensory fibres (Ren et al., 2015).

Reviews with coverage of purinergic signalling and cutaneous pain are available (Zhu and Lu, 2010; Burnstock, 2016c).

### Dermatitis

In irritant dermatitis, there is a pathogenic role for keratinocyte-derived ATP. The necrosis produced by the chemical irritant croton oil was prevented by the pre-treatment with A438079, a selective P2X7R antagonist (Zanin et al., 2015). Topical application of A_2A_ antagonists prevents radiation dermatitis (Perez-Aso et al., 2016).

### Skin Cancer

Different subtypes of P1R and P2R are involved in skin cancer, playing roles in differentiation, apoptosis and proliferation. The roles of purinoceptors are complicated because there are multiple receptor subtypes on the same cell, which can have opposing effects. For example, P2Y_2_R mediate an increase in tumour cell numbers, while P2Y_1_, P2X5, and P2X7R mediate a decrease in cell numbers. In cancers of the skin, proliferation outweighs apoptotic cell death.

UV light is a stimulus for the genesis of cutaneous cancer, including basal and squamous cell carcinoma as well as melanoma, where the UV-B component has the most severe effects. UV-B irradiation decreases the amount of P2X1, P2Y_2_, and P2X7R, which contributes to the malignant transformation of keratinocytes (Ruzsnavszky et al., 2011).

#### Basal and Squamous Cell Carcinomas

Basal cell and squamous cell carcinoma are tumours that generally occur after the age of 50, squamous cell carcinoma being the more common and aggressive of the two. Nucleoside analogues reduced basal cell carcinoma growth. A human cutaneous squamous cell (epidermal) carcinoma cell line, A431, express P2R that induce increases in [Ca^2+^]_i_. ATP-stimulated A431 cells induced production of inositol-trisphosphate, suggesting the involvement of P2YR. P2X5 and P2YR were strongly expressed on both squamous and basal cell carcinomas. P2X7R are expressed in apoptotic cells in superficial multifocal and infiltrative cells and in the necrotic centre of nodular basal cell carcinomas. Expression of P2Y_1_R was confined to the stroma surrounding tumours. P2Y_4_R were present only in basal cell carcinomas. ATP and UTP at low concentrations induced an increase in the number of A431 cells, while high concentrations substantially decreased cell numbers.

ATP via P2X7R caused apoptosis of A431 cells and UTP and adenosine (after ATP breakdown) also induced cell death (Völkl et al., 2008). 2′(3′)-*O*-(4-Benzoylbenzoyl) ATP, a potent P2X7R agonist, reduced skin carcinoma and papilloma and formation (Fu et al., 2009). ADA in saliva was shown to be a diagnostic marker of tongue squamous cell carcinoma (Rai et al., 2011).

#### Melanoma

Malignant melanoma, which is highly metastatic, is derived from melanocytes. ATP inhibited the growth of both human and animal melanoma cells *in vivo*. CD39 is over-expressed in differentiated human melanomas. Expression of P2X7R was increased in superficial spreading melanoma patients, which were later shown to be functional and may be a therapeutic target for melanoma therapy (White et al., 2005). A low pH environment as seen in solid tumours induced ATP release from B16 melanoma cells, which increases proliferation via P2X7R. Oxidised ATP, a P2X7R antagonist, inhibited tumour growth (Hattori et al., 2012). P2Y_1_, P2Y_2_, and P2Y_6_R mRNA and protein expression was observed in human melanomas. The presence of P2Y_1_ and P2X7R, which had been suggested to be therapeutic targets for melanoma treatment using ATP, was demonstrated by immunohistochemistry (White et al., 2009). The release of ATP from murine B16 melanoma cells was shown to upregulate CD39 expression on regulatory T cells. Dying tumour cells release ATP, which accumulates at high concentrations and acts as an immune danger signal, although it can also kill adjacent tumour cells directly via P2X7R (Feng et al., 2011). Accelerated melanoma tumour progression in mice lacking P2X7R has been reported (Adinolfi et al., 2015). γ-Irradiation that induces arrest in tumour cell growth and death, induced P2X7R-dependent release of ATP from B16 cells (Ohshima et al., 2010).

In an animal model of melanoma, tumour growth was reduced in CD73 KO mice (Yegutkin et al., 2011). Implanting B16 cells into CD73 KO mice, thereby decreasing the production of adenosine, resulted in reduced tumour growth (Ring et al., 2011). Adenosine potentiated the *in vivo* actions of chemotherapeutic agents. Antimetastatic therapies based on inhibition of A_1_R activation have been suggested. A_1_, A_2A_, A_2B_ and A_3_R subtypes are expressed in the A375 human malignant melanoma cell line. A_3_R activation resulted in growth inhibition of melanoma cells (Morello et al., 2011). However, another study claimed that adenosine, acting through A_3_R, induced cell proliferation of human malignant melanoma C32 cells (Soares et al., 2012). A_2B_R antagonists impaired IL-8 production that is raised in malignant melanoma patients, while A_3_R antagonists decreased vascular endothelial growth factor that promotes human carcinoma cell angiogenesis and metastasis (Merighi et al., 2009). Adenosine is a potent immunoregulatory factor modulating cytotoxic activity and cytokine production of anti-melanoma specific T cells.

In conclusion, P2Y_2_R mediate proliferation, P2X5R mediate differentiation (i.e., are antiproliferative) and P2X7R mediate cell death. Therefore, P2Y_2_R antagonists and P2X5 and P2X7R agonists have therapeutic potential for the treatment of skin cancer.

## Musculoskeletal Diseases

Reviews that include discussion of the pathophysiology of musculoskeletal diseases have been published (Burnstock et al., 2013; Young et al., 2013; Agrawal and Gartland, 2015; Jørgensen et al., 2015; Orriss et al., 2016). There are multiple purinoceptor subtypes expressed by bone and cartilage, which are potential targets for original therapeutic strategies to inhibit bone resorption in osteoporosis, rheumatoid arthritis (RA), periodontitis and tumour-induced osteolysis.

### Muscular Dystrophy

ATP was used early to treat myopathies, although the mechanism of action was not known. Allopurinol has been used for the treatment of Duchenne muscular dystrophy (DMD). It counteracts low purine nucleotide degradation levels that occur in Duchenne muscle, but chronic administration of allopurinol failed to improve DMD symptoms. Lymphoblastoid cells from DMD patients were found to be highly sensitive to ATP stimulation.

The *mdx* model of DMD, lacking the dystrophin protein, mimics muscle damage and subsequent regeneration. Sequential expression of P2X5, P2Y_1_ and P2X2R was described during muscle regeneration in the *mdx* model (Ryten et al., 2004). It was claimed recently that a P2Y_2_R antagonist may ameliorate cardiomyopathy in DMD (De Oliveira Moreira et al., 2017). P2Y_1_R were expressed on infiltrating immune cells. A purinergic dystrophic phenotype was seen during the earliest stage of developing dystrophic muscle. Dystrophic myoblasts express P2X4 and P2X7R proteins and it was claimed that antagonists to these receptors may be of potential therapeutic benefit. ATP signalling is altered in muscular dystrophy, but was partly recovered after nifedipine treatment (Valladares et al., 2014). It was claimed that dihydropyridines may be used as a therapeutical tool to reduce muscle damage observed in dystrophic muscles. It was suggested that P2XR antagonists alter the adaptive immune component in the muscle infiltrates in DMD and are a promising therapeutic approach to treat DMD (Gazzerro et al., 2015).

Myofascial pain is a feature of DMD and ATP stimulates myofascial nociceptors. P2X7R upregulation occurs in dystrophic *mdx* mouse muscles and treatment with P2X7R antagonists slows the progression of DMD (Sinadinos et al., 2015). Sensitivity to ATP is higher, release of ATP is greater and expression of P2Y_2_R is increased, but P2Y_1_R expression is decreased in *mdx* mice. A review about purinergic receptors in DMD was published (Krasowska et al., 2014).

### Myasthenia Gravis (MG)

Myasthenia gravis is an autoimmune disease, which affects the neuromuscular junction. Lack of neuronal A_2A_R-mediated Ca_v_ 1 (L-type) influx causes tetanic failure in MG (Noronha-Matos et al., 2011). It was suggested that the adenosinergic pathway was dysfunctional in autoimmune MG and that stimulation of CD73 activity and A_2A_R may have therapeutic potential for MG (Oliveira et al., 2015).

### Fibromyalgia

Fibromyalgia (a musculoskeletal disease) is characterised by allodynia, as well as mood disorders. In fibromyalgia patients, there are decreased levels of ATP in platelets perhaps contributing to the pathogenesis of the disease (Bazzichi et al., 2008).

### Osteoporosis

Osteoporosis, characterised by bone mass loss (bone mineral density decrease), leads to a high risk of bone fracture. The P2X7R is considered to be important in relation to the treatment of osteoporosis (Kvist et al., 2014). P2X7R plays a critical role in both cortical and cancellous bone mass augmentation and were shown to stimulate cancellous and periosteal bone formation and inhibit cancellous bone resorption during growth. Single nucleotide polymorphisms of the P2X7R gene are associated with the risk of fractures, decrease in bone mineral density and osteoporosis. P2X7R are involved in the formation of human osteoclasts. P2X7R antagonists are being considered for the treatment of osteoporosis and with remodelling disorders, where bone mass is reduced (Jørgensen et al., 2011). Skeletal pain accompanies osteoporosis and P2X2/3R might have a role in osteoporosis patients under a high bone turnover state (Iba and Yamashita, 2016). P2X5R may be a therapeutic target for treatment for inflammatory bone loss (Kim et al., 2017). Polymorphisms in the P2X4 and P2X7R genes (Husted et al., 2013) and the Leu46Pro polymorphism of the human P2Y_2_R gene were linked to bone mineral density and the risk of osteoporosis in Dutch fracture patients (Wesselius et al., 2013). It was suggested that clopidogrel, a P2Y_12_R antagonist used for stroke and thrombosis, increases the risk of fractures in osteoporotic patients (Jørgensen et al., 2012). P2Y_13_R antagonists have also been proposed for the treatment of osteoporosis (Wang N. et al., 2013).

Adenosine receptors are involved in osteoporosis (McPhee and Whiting, 1989). *Trans*-differentiation of osteoblasts to adipocytes, which involves A_2B_R, may contribute to the pathogenesis of osteoporosis (Rayalam et al., 2011). P1R might be targets for treating osteoporosis and other diseases characterised by excessive bone turnover.

### Osteoarthritis (OA)

Osteoarthritis is a degenerative joint disease as a result of wear of the articular cartilage due to abnormal load to the joint or from infection, trauma or due to ageing. The pain from OA is due largely to inflammation. Increased ATP levels and 5′-nucleotidase activity are present in osteoarthritic joint synovial fluid, compared to the joints from RA patients, particularly from osteoarthritic patients where deposition of calcium-containing crystals were also present. ATP contributes to pathologic mineralisation in articular cartilage and therefore P2R antagonists might provide therapeutic tools for crystal-associated arthritis (Costello et al., 2011). ATP levels in knee synovial fluid of patients with OA are related to pain intensity (Kumahashi et al., 2011). P2X3 and P2X2/3R play an important role in the development of articular hyperalgesia of arthritic joints (Teixeira et al., 2016). In the long-term complications following total hip arthroplasty, different polymorphic variants of the P2X7R are associated with high or reduced periprosthetic osteolysis (Mrazek et al., 2010). There are elevated concentrations of ATP in the synovial fluid of dogs with OA (Torres B.T. et al., 2016). Purinergic signalling, via P2R, produces calcium oscillations in migratory chondrogenic progenitor cells isolated from OA cartilage (Matta et al., 2015). Platelets promote cartilage repair and chondrocyte proliferation via release of ADP in a rodent model of OA, suggesting that P2Y_1_ or P2Y_12_R agonists may be useful for treatment of OA (Zhou Q. et al., 2016). Treatment with the selective P2X7R antagonist, AZD9056, produced pain-relieving and anti-inflammatory effects in rats with OA (Hu H. et al., 2016).

Adenosine signalling is also involved in OA. Adenosine, produced following breakdown of ATP, is released from chondrocytes and contributes to tissue damage in arthritic conditions. The regulation of LPS-induced IL-6 release involves A_2A_R, indicating that adenosine has a regulatory role in controlling osteoclastogenesis and inflammation. Electromagnetic field stimulation up-regulates A_2A_R in synovial fibroblasts and adenosine, acting through both A_1_ and A_2A_R, had anti-inflammatory activity to control joint inflammation (De Mattei et al., 2009). A_2A_ and A_3_R agonists modulate prostaglandin E_2_ and cytokine release in human osteoarthritic fibroblasts (Ongaro et al., 2012). Adenosine receptors also mediate regulation of inflammatory responses in human synoviocytes (Varani et al., 2010b). A_2A_R agonists are used to reduce joint destruction due to septic arthritis (Cohen et al., 2004). A_2A_R deletion resulted in the development of OA in mice, suggesting that A_2A_R agonists might be a target for the treatment of OA (Corciulo et al., 2017).

### Rheumatoid Arthritis (RA)

The potential involvement in RA of purinergic signalling was recognised first in the 1990’s when concentrations of adenosine, after released ATP was broken down, were reduced in synovial fluid in RA compared to OA. Increased levels of adenosine as a treatment for RA was suggested. UTP and ATP activate calcium-mobilising P2UR to synergistically act with IL-1 to stimulate release of prostaglandin E_2_ from human rheumatoid synovial cells. Hypotonic stress promotes ATP release and cell proliferation via transient receptor potential vanilloid 4 activation in RA rat synovial fibroblasts (Hu et al., 2017).

IL-1β is a proinflammatory cytokine that substantially contributes to the progression of RA. ATP, through P2X7R, induced increased levels of IL-1β in RA patient blood samples compared to control samples. Mononuclear cells from these patients were more sensitive to stimulation by ATP, possibly because of P2X7R genetic polymorphism (Portales-Cervantes et al., 2012). P2X7R are involved in the pathogenesis of RA and systemic lupus erythematosus (Portales-Cervantes et al., 2010). P2X7R mRNA and protein are expressed in human rheumatoid synoviocytes. Studies of arthritis animal models suggest an *in vivo* role for the P2X7R in the progression of this inflammatory disease. There was a lower incidence and reduced severity of the symptoms of arthritis induced by anti-collagen treatment in P2X7R KO mice. Therefore targeting the P2X7R may be a potential treatment for RA. P2X7R antagonists in the collagen-induced animal model of RA reduced destruction of peripheral inflammatory tissue. In a later study, it was claimed that AZD9056, a P2X7R antagonist, was not effective against RA (Keystone et al., 2012). P2X7R antagonists are being investigated for clinical use against inflammatory joint pain (Beswick et al., 2010). A multicentre, double-blind, placebo-controlled clinical trial showed that ATP infusions reduced inflammation and disease symptoms in patients with RA (Bours et al., 2010).

Bovine chondrocytes express P2X1 and P2X3R and following stimulation there was release of inflammatory mediators. Therefore antagonists to these receptors may be therapeutically useful for articular cartilage resorption and diseases involving inflammation. There was an increased platelet response to ADP in RA patients (Mac Mullan et al., 2010).

Sympathetic nerves mediate proinflammatory responses during the initial phase of arthritis induced by type II collagen, probably via cytokines such as interferon-γ released in response to the sympathetic cotransmitters ATP and NA (Straub et al., 2008). α,β-MeATP-sensitive P2XR (probably P2X3) are expressed on rat knee joint peripheral nociceptive afferent fibres and the increased ATP levels in damaged and inflamed tissues, may contribute to nociception and pain. P2X3R were found on nociceptive sensory fibres in lumber facet joints, where low back pain originates (Ishikawa et al., 2005). Changes in P2X3R expression on DRG neurons that label isolectin B4 were seen following the induction of RA (Averill et al., 2008). P2X3R expressed on trigeminal ganglia also contribute to orofacial pressure pain in monoarthritis of the temporomandibular joint. Plasma extravasation in the rat knee joint induced by bradykinin was enhanced by ATP, released as a cotransmitter from sympathetic nerves. Intravenous guanethidine, which inhibits release of sympathetic cotransmitters, proved to be effective in RA patients. *Uncaria tomentosa* extract affects the metabolism of adenine nucleotides and has been suggested as an adjuvant to treat arthritis (Castilhos et al., 2015).

The evidence showing a role for adenosine in RA has been reviewed (Varani et al., 2010a). Increased activity of ADA was seen in synovial fluid from patients with RA as well as in rheumatoid synovial fibroblasts (Nakamachi et al., 2003). The ImmuKnow assay might effectively identify RA patients that are more at risk of developing infections (Akimoto et al., 2013). Signalling via the A_2A_R caused modification of the cytokine milieu in RA (Masahiro et al., 2003). Over-expression of A_3_R was observed in peripheral blood mononuclear cells of RA patients. Patients with RA had greater expression A_3_R in the synovium (Stamp et al., 2012). There are increased levels of A_2A_ and A_3_R on the lymphocytes and neutrophils of RA patients. In the dorsal horn of rats with induced RA, A_1_R agonists decreased activation of *c-fos* and astrocytes. In a later study of the adjuvant-induced monoarthritis model an A_3_ specific agonist prevented bone resorption. ATP, working via A_2_R, also reduced joint injury. A phase II clinical trial provided evidence for A_3_R agonists as a treatment for RA (Fishman et al., 2008; Silverman et al., 2008). CGS 21680, an agonist of the A_2A_R, reduced progression of murine type II collagen-induced arthritis (Mazzon et al., 2011). A_2A_R agonists ameliorate adjuvant-induced arthritis in rats (Vincenzi et al., 2013). Methotrexate (MTX) is often used to treat RA. In human joints with inflammatory disease, MTX treatment involved A_2A_R. Adenosine via A_2B_R prevented MTX-induced inhibition of osteoclast bone destruction in arthritis induced by adjuvant (Teramachi et al., 2011). In RA patients treated with MTX, studies of polymorphisms of the genes involved in adenosine release concluded that genotyping may help identify patients who would most benefit from MTX treatment (Wessels et al., 2006). Anti-tumour necrosis factor-α has also been used to treat RA, but it raises the risk of reactivating tuberculosis. ADA assay is a specific and sensitive test for the quick diagnosis of rheumatoid effusions (Zakeri et al., 2012).

### Tooth Pain

There are many sensory nerves, originating in the trigeminal ganglia, expressing P2X3R in tooth pulp and ATP is released from odontoblasts in response to mechanical stimulation to act on these receptors, resulting in pain (Shibukawa et al., 2015). LPS-induced pulp inflammation increased the expression of P2XR in trigeminal sensory nerves (Chen et al., 2014). Mechanical or cold stimulation of odontoblast processes in dentin tubules, results in ATP release and dental pain (Liu X. et al., 2015). Therefore P2X3R antagonists may be therapeutically useful to reduce toothache.

### Bone Cancer Pain

Bone metastases, common in prostate and breast cancer patients, may cause substantial bone loss and pain. Purinergic signalling involvement in bone cancer was initially reported in the 1990’s when P2U (i.e., P2Y_2_/P2Y_4_) receptors were cloned from osteoclastoma. The expression of P2Y_2_R from human osteoclasts from bone giant cell tumour was later reported. Butyl benzyl phthalate, which interferes with mammalian ion channel receptors, inhibited ATP-induced cell proliferation via P2XR in human osteosarcoma HOS cells (Liu and Chen, 2010). ATP was used in autologous bone marrow transplantation for removing residual tumour cells. In mice, a type of human apyrase, APT102, in addition to aspirin disrupts bone metastasis (Uluçkan et al., 2008). Release of ATP from tumour cells further stimulates osteoclast formation and activity, contributing to bone destruction that often happens around tumour metastases. Bisphosphonates, used to treat osteoporosis, treat bone cancer and may involve apoptosis induced by ApppI, an ATP analogue produced by bisphosphonates (Sillero et al., 2009).

Purinergic signalling plays a role in bone cancer pain. Bone pain can be relieved by radiation therapy, which may be related to the Ca^2+^-signalling cascade, mediated by P2X6R. In a mouse model of cancer pain, increased expression of P2X3R on calcitonin gene-related peptide immunoreactive DRG neurons during tumour growth, it was claimed that ATP had a role in cancer-related pain (Liu M. et al., 2013). In rats, systemic inhibition of P2X3 and P2X2/3R with AF-353 strongly attenuated bone cancer pain-related behaviour (Kaan et al., 2010). In mice, administration of A-317491, a selective P2X3 and P2X2/3R antagonist, attenuated the early stages of bone pain in cancer (Hansen et al., 2012). Functional up-regulation of P2X3R has been described in DRG of bone cancer pain in a rat model (Wu et al., 2012).

In P2X7R KO mice, bone cancer pain-related behaviours had an earlier onset (Hansen et al., 2011). Most human osteosarcomas expressed P2X7R isoforms A and B (Giuliani et al., 2014) and P2X7R are involved in cancer-induced bone pain and P2X7R antagonists were suggested as a useful analgesic target (Falk et al., 2015). P2Y_1_R signalling in the DRG and spinal cord may mediate pain from bone cancer (Chen et al., 2012). Activation of K_ATP_ channels at the spinal cord level reduces pain associated with bone cancer (Xia et al., 2014). Stimulation by AMP-activated protein kinase suppresses neuroinflammation and reduces bone cancer pain (Song et al., 2015).

### Myeloma

Multiple myeloma (cancer of plasma cells) involves osteolytic bone lesions, due largely to enhanced osteoclast activity. A_2_R may be therapeutically useful to treat and prevent multiple myeloma-induced bone disease as activation of A_2A_R reduces osteoclast function, while activation of A_2B_R stimulates osteoblast differentiation (He et al., 2012). 8-Amino-adenosine is another possible therapeutic compound for the treatment of multiple myeloma.

### Severe Combined Immunodeficiency

A major cause of severe combined immunodeficiency are genetic defects in the ADA gene. Lack of ADA causes accumulation of adenosine. Bone defects as a result of reduced osteoclastogenesis together with a defect in osteoblast function leading to low bone formation were seen in about a half of early-onset ADA-deficient patients (Sauer et al., 2009). Further, the microenvironment of bone marrow in ADA KO mice had a lower ability to support haematopoiesis. In ADA KO mice, treatment with gene therapy, bone marrow transplantation or enzyme replacement, led to a full recovery. ADA-transduced hematopoietic stem cell gene therapy also enhanced the growth of children with this disease.

### Dwarfism (Achondroplasia)

This is a congenital dysplasia of the skeleton as a result in a mutation in the gene encoding fibroblast growth factor receptor type 3 (FGR3). Ap_4_A diminished the expression of the achondroplasic FGFR3 receptor and P2Y_1_, P2Y_2_, P2Y_6_, and P2Y_11_R are expressed by achondroplasic chondrocytes mediating the action of Ap_4_A (Guzmán-Aránguez et al., 2008). Ap_4_A reversed the morphological changes supporting a therapeutic role for Ap_4_A as a possible treatment of dwarfism (Huete et al., 2011).

### Paget’s Disease

There is an increase in osteoclast numbers in Paget’s disease, leading to an increase in bone resorption and a high turnover of bone. Bisphosphonates have been employed as a treatment for Paget’s disease and P2X7 antagonists have also been considered (Agrawal et al., 2010).

### Ossification of the Posterior Longitudinal Ligament of the Spine

This disease causes neurological damage as a result of ectopic bone formation in spinal ligaments. In this disease, extracellular ATP in ossification of cell cultures of the posterior longitudinal ligament of the spine (OPLL) is increased. P2Y_1_R are highly expressed in OPLL cells. Mechanical stress and ATP increase the levels of osteopontin and alkaline phosphatase mRNA in OPLL cells, effects that can be inhibited by MRS2179, a selective P2Y_1_R antagonist. Over-expression of P2Y_1_R in OPLL-induced mineralisation resulted in ectopic bone formation in the spinal ligament cells of patients with OPLL (Tanaka et al., 2011).

## Concluding Comments

Clinical interventions involving purinergic signalling are just beginning. However, the beginning and future of purinergic compounds for the treatment of a wide range of diseases is described in this review. P2Y_12_R antagonists, such as clopidogrel and ticagrelor, are currently in wide use for stroke and thrombosis, as are P2Y_2_R agonists for dry eye and A_1_R agonists for supraventricular tachycardia. The use of P2X7R antagonists for the treatment of inflammatory diseases is promising, but the presence of polymorphic variations of this receptor is holding up the development of selective antagonists appropriate for each disease. P2X3R antagonists are in clinical trials for use against visceral pain, chronic cough and hypertension. A_2A_R agonists are in use for the treatment of PD, and perhaps soon in wider use. P2X1R antagonists are being investigated for treatment of bladder disorders and hypertension, while P2X4, P2X7 and A_3_R antagonists are being explored for neuropathic pain. Treatments with inhalation of ectonucleotidases to alter the balance of ATP and adenosine and inhibitors of ATP release, are also a therapeutic approach being explored. The development of novel purinergic compounds by medicinal chemists that are orally available and stable *in vivo* would be a significant advantage in developing therapeutic approaches, including centrally penetrant P2X7R antagonists (Able et al., 2011).

The majority of the therapeutic approaches for many heart disorders based on purinergic signalling manipulation are not fully understood yet and strategies to overcome the side-effects of treatment need to be considered. The pathophysiological roles of purinergic signalling in blood vessels are clearer and it plays an important role in controlling vascular tone and remodelling. Immunologic factors related to purinergic signalling are attracting more attention and should be considered (Cekic and Linden, 2016). Human embryonic stem cells are able to self-renew and have the potential to differentiate into different cell types, including cardiovascular progenitor cells. This system of differentiation is being investigated for cardiac regenerative therapy (Huang et al., 2016). The single nucleotide polymorphisms in purinergic receptor genes and their association with diseases are being explored for potential use as diagnosis biomarkers (see Caseley et al., 2014). Purinoceptors modulate neural stem cell proliferation, differentiation, migration and cell death and could be therapeutic approaches for the treatment of neurological and psychiatric illnesses (Illes and Rubini, 2017). MicroRNAs modulating purinergic signalling are gaining interest as potential original therapeutic targets and disease biomarkers (Ferrari et al., 2016a).

Although still in its infancy, clinical use of purinergic compounds has started. Several relevant pharmacological interventions are currently in clinical use. The lack of more established purinergic therapies may be due to there being relatively few receptor subtype-specific agonists and antagonists that are both effective and stable *in vivo* (see Jacobson and Muller, 2016). In some situations, a degree of redundancy is present, with several different subtypes of receptor mediating similar functional effects. Purinergic signalling is implicated in multiple disorders and therefore offers many potential future therapeutic targets. It should be noted, however, that since most purinoceptors are ubiquitous, to selectively target specific cell types may prove to be a challenge. As well as the development of selective agonists and antagonists, therapeutic strategies will probably include compounds that control P2R expression, inhibitors of extracellular ATP breakdown and inhibitors or enhancers of ATP transport. Understanding the interactions of purinergic signalling with other established signalling systems will be necessary.

## Author Contributions

The author confirms being the sole contributor of this work and approved it for publication.

## Conflict of Interest Statement

The author declares that the research was conducted in the absence of any commercial or financial relationships that could be construed as a potential conflict of interest. The reviewer SS and handling Editor declared their shared affiliation.
